# Optimized CRISPR-Cas9 Genome Editing for *Leishmania* and Its Use To Target a Multigene Family, Induce Chromosomal Translocation, and Study DNA Break Repair Mechanisms

**DOI:** 10.1128/mSphere.00340-16

**Published:** 2017-01-18

**Authors:** Wen-Wei Zhang, Patrick Lypaczewski, Greg Matlashewski

**Affiliations:** Department of Microbiology and Immunology, McGill University, Montreal, Canada; University at Buffalo

**Keywords:** CRISPR-Cas9, chromosomal translocation, co-CRISPR targeting, homology-directed repair, *Leishmania*, MMEJ, microhomology-mediated end joining, Rad51, double-strand break repair, genome editing, miltefosine transporter, multiple gene family

## Abstract

CRISPR-Cas9-mediated genome editing has recently been adapted for *Leishmania* spp. parasites, the causative agents of human leishmaniasis. We have optimized this genome-editing tool by selecting for cells with CRISPR-Cas9 activity through cotargeting the miltefosine transporter gene; mutation of this gene leads to miltefosine resistance. This cotargeting strategy integrated into a triple guide RNA (gRNA) expression vector was used to delete all 11 copies of the A2 multigene family; this was not previously possible with the traditional gene-targeting method. We found that the *Leishmania donovani* rRNA promoter is more efficient than the U6 promoter in driving gRNA expression, and sequential transfections of the oligonucleotide donor significantly eased the isolation of edited mutants. A gRNA and Cas9 coexpression vector was developed that was functional in all tested *Leishmania* species, including *L. donovani*, *L. major*, and *L. mexicana*. By simultaneously targeting sites from two different chromosomes, all four types of targeted chromosomal translocations were generated, regardless of the polycistronic transcription direction from the parent chromosomes. It was possible to use this CRISPR system to create a single conserved amino acid substitution (A189G) mutation for both alleles of* RAD51*, a DNA recombinase involved in homology-directed repair. We found that RAD51 is essential for *L. donovani* survival based on direct observation of the death of mutants with both *RAD51* alleles disrupted, further confirming that this CRISPR system can reveal gene essentiality. Evidence is also provided that microhomology-mediated end joining (MMEJ) plays a major role in double-strand DNA break repair in *L. donovani*.

**IMPORTANCE** *Leishmania* parasites cause human leishmaniasis. To accelerate characterization of *Leishmania* genes for new drug and vaccine development, we optimized and simplified the CRISPR-Cas9 genome-editing tool for *Leishmania*. We show that co-CRISPR targeting of the miltefosine transporter gene and serial transfections of an oligonucleotide donor significantly eased isolation of edited mutants. This cotargeting strategy was efficiently used to delete all 11 members of the A2 virulence gene family. This technical advancement is valuable, since there are many gene clusters and supernumerary chromosomes in the various *Leishmania* species and isolates. We simplified this CRISPR system by developing a gRNA and Cas9 coexpression vector which could be used to delete genes in various *Leishmania* species. This CRISPR system could also be used to generate specific chromosomal translocations, which will help in the study of *Leishmania* gene expression and transcription control. This study also provides new information about double-strand DNA break repair mechanisms in *Leishmania*.

## Abstract (precis)

*Leishmania* parasites cause human leishmaniasis. To accelerate characterization of *Leishmania* genes for new drug and vaccine development, we optimized and simplified the CRISPR-Cas9 genome-editing tool for *Leishmania*. We show that co-CRISPR targeting of the miltefosine transporter gene and serial transfections of an oligonucleotide donor significantly eased isolation of edited mutants. This cotargeting strategy was efficiently used to delete all 11 members of the A2 virulence gene family. This technical advancement is valuable, since there are many gene clusters and supernumerary chromosomes in the various *Leishmania* species and isolates. We simplified this CRISPR system by developing a gRNA and Cas9 coexpression vector which could be used to delete genes in various *Leishmania* species. This CRISPR system could also be used to generate specific chromosomal translocations, which will help in the study of *Leishmania* gene expression and transcription control. This study also provides new information about double-strand DNA break repair mechanisms in *Leishmania*.

## INTRODUCTION

Leishmaniasis is a vector-borne disease caused by the protozoan parasite species of the genus *Leishmania*. Depending on the species, *Leishmania* infection can cause mild self-healing cutaneous leishmaniasis (CL), disfiguring mucocutaneous leishmaniasis (MCL), or fatal visceral leishmaniasis (VL), which is the second-deadliest parasitic disease after malaria (1, 2). Approximately 1 billion people worldwide are at risk of infection, and more than 1.3 million new infections occur each year. Despite decades of research, there is still no vaccine, and treatment of leishmaniasis relies on drugs which are expensive, toxic, and are at risk for resistance development (1, 2).

The *Leishmania* genome contains over 8,000 genes, and most of these genes have unknown functions (3–6). Since its introduction to *Leishmania* research nearly 3 decades ago, the traditional gene targeting method involving homologous recombination using antibiotic selection marker genes has greatly contributed to the understanding of *Leishmania* biology and pathogenesis. This homologous recombination method is, however, time-consuming, limited by available antibiotic selection markers, and not well-suited for introducing point mutations and other genome-editing tasks. Thus, a simpler yet more efficient and versatile genome-editing method is required to accelerate the characterization of *Leishmania* genes for new drug target identification and vaccine development.

We have recently developed vectors expressing the Cas9 nuclease and guide RNA (gRNA) for *Leishmania* spp., and we demonstrated that CRISPR-Cas9 is an effective genome engineering tool for *L. donovani* (7). It was revealed that *L. donovani* mainly uses homology-directed repair (HDR) and microhomology-mediated end joining (MMEJ) to repair Cas9-generated double-strand DNA (dsDNA) breaks and that the nonhomologous end-joining (NHEJ) pathway appears to be absent in *L. donovani*. MMEJ resulted in deletion mutations ranging from 10 to more than 3,000 bp. The activity of different gRNAs can vary significantly. The use of an oligonucleotide donor, antibiotic selection marker donor, and double gRNA expression vector greatly improved the precision and efficiency of CRISPR-Cas9-mediated genome editing (7).

In this study, we further optimized and simplified CRISPR-Cas9-mediated genome editing in *Leishmania* through several approaches. Single vectors capable of expressing both the gRNA and Cas9 nuclease were developed which were functional in all tested *Leishmania* species, including *L. donovani*, *L. major*, and *L. mexicana*. Sequential transfections of a gene-editing oligonucleotide donor significantly eased the isolation of the edited mutants. Cotargeting the miltefosine transporter gene (*MT*) followed by miltefosine selection greatly increased the efficiency of editing a second target gene in parallel. This cotargeting strategy, integrated into a multiple gRNA expression vector, was used to delete all 11 members of the A2 gene family in *L. donovani*, an unattainable task with the traditional gene targeting method (8). We generated specific chromosomal translocations by simultaneously targeting sites from different chromosomes. We demonstrated that RAD51, a DNA recombinase involved in HDR, is essential for *L. donovani*. However, analysis involving the inhibition of RAD51 strongly suggested that MMEJ plays the major role in double-strand DNA break repair in *Leishmania*.

## RESULTS AND DISCUSSION

### Sequential transfections of an oligonucleotide donor significantly improve CRISPR-Cas9 gene-editing efficiency.

**FIG 1:**
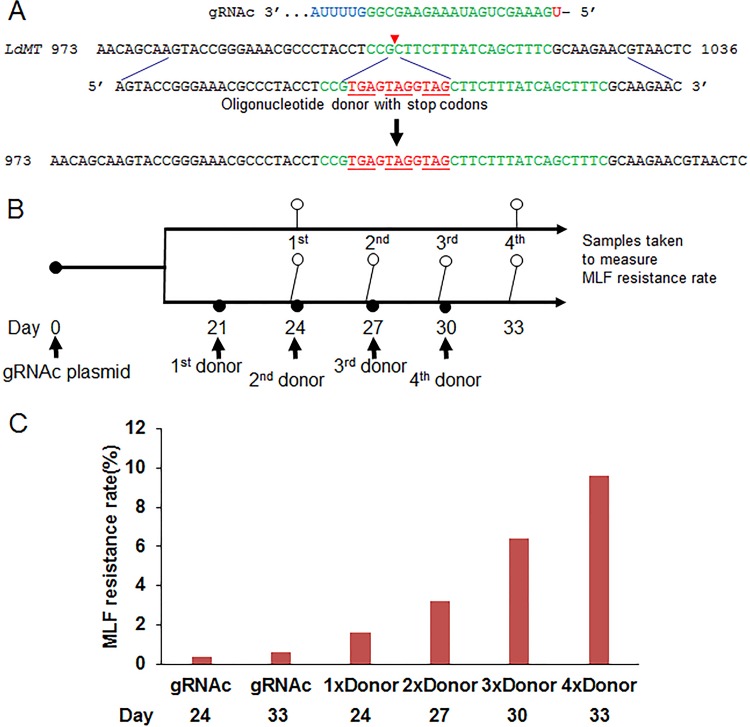
Sequential transfections of oligonucleotide donors significantly improve CRISPR-Cas9 gene-editing efficiency. (A) Gene-editing strategy by providing the gRNAc-directed Cas9 cleavage site in the *LdMT* locus with the 61-nt single-strand oligonucleotide donor containing stop codons. (B) Oligonucleotide donor transfection schedule and time points for sampling miltefosine (MLF) resistance rates. (C) The MLF resistance rates of gRNAc-targeted *L. donovani* cells after sequential transfections of the oligonucleotide donor. The MLF resistance rates were determined in 96-well plates by limiting dilution culture at 27°C for 2 to 4 weeks, as previously described (7). These are representative data of three independent experiments.

We previously showed that the addition of an oligonucleotide donor improved CRISPR-Cas9 gene disruption efficiency in *L. donovani* (7). In some cases, however, a gene-editing task, such as the introduction of an epitope tag or a point mutation, may involve a site where only low-activity gRNAs can be designed, such as the gRNAc-targeting site in the *L. donovani* miltefosine transporter gene *MT* (7). We therefore wanted to determine whether sequential transfections of an oligonucleotide donor containing 25-nucleotide-long flanking homologous sequences to the Cas9 break site would increase the gene-editing efficiency and improve the isolation of the edited mutants (Fig. 1A; see also Data Set S1 in the supplemental material). The *MT* gene was selected for evaluating CRISPR-Cas9 gene-editing efficiency (frequency), since mutations (insertions, deletions, and selected point mutations) in the *MT* gene lead to survival (resistance) in the presence of miltefosine (7). Twenty-one days following transfection of vectors expressing gRNAc and Cas9 in *L. donovani* (21 days is the time required to select for cells with stable expression), sequential transfections with an oligonucleotide donor containing stop codons were carried out every 3 days for a total of four transfections (Fig. 1B). The miltefosine resistance rate was determined 3 days after each oligonucleotide donor transfection. As shown in Fig. 1C, multiple transfections increased the miltefosine resistance rate significantly, compared to gRNA expression alone. At the end of the fourth donor oligonucleotide transfection, the final miltefosine resistance rate in these gRNAc-expressing cells increased from 0.4% without a donor to nearly 10%, representing a 25-fold increase. This demonstrated that sequential transfections of an oligonucleotide donor can significantly improve CRISPR-Cas9 gene editing (oligonucleotide donor-directed repair) efficiency to make it easier to isolate the edited mutants. Since the growth rate can be altered in edited mutants, it is necessary to clone transfectants into 96-well plates within 2 days after the third or fourth donor transfection. We have successfully used this strategy to generate *L. major* and *L. mexicana *centrin gene null mutants (data not shown) and the single-amino-acid substitution mutant for *L. donovani* RAD51 (see below).

10.1128/mSphere.00340-16.1 DATA SET S1  *L. donovani* miltefosine transporter gene (*LdMT*; LdBPK_131590.1) sequence and the sequences of gRNA guides, oligonucleotide donors, and primers used to generate and detect *LdMT* mutants. The locations and directions of gRNA guides and primers in the *LdMT* gene are indicated (underlined with an arrow). Some of these primers had a restriction enzyme site added to their 5′ end to facilitate cloning of the PCR product. Download Data Set S1, DOCX file, 0.04 MB. Copyright © 2017 Zhang et al. 2017 Zhang et al. This content is distributed under the terms of the Creative Commons Attribution 4.0 International license.

### Deletion of the multicopy A2 gene family by cotargeting the miltefosine transporter (*MT*) gene and selection with miltefosine.

**FIG 2:**
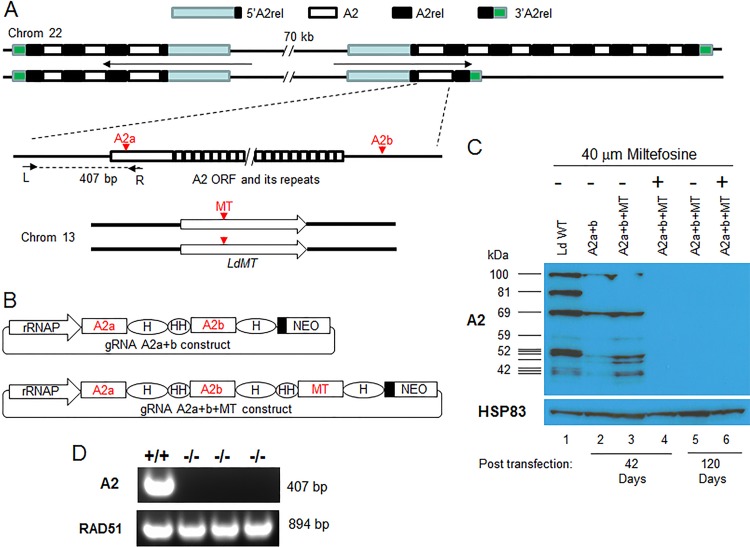
Deletion of the multicopy A2 family genes and increased efficiency through coselection for parasites with CRISPR-Cas9 activity. (A) Schematic drawing of A2-A2rel gene cluster loci in *L. donovani* 1SCl2D chromosome 22 and the A2 deletion coselection strategy through cotargeting the *LdMT* (miltefosine transporter gene). There are two nonidentical A2-A2rel gene clusters with outward transcription directions in chromosome 22. The 1SCl2D strain has at least 11 copies of A2 genes of various sizes, which alternate with A2rel genes and are flanked by 5′ A2rel and 3′ A2rel genes. The A2 and MT gRNA-targeting sites and primers used to verify A2 gene deletion are indicated. Note that this putative A2-A2rel gene cluster is based on our previous A2-targeting study (8) plus recent unpublished PacBio genome sequencing data. Because of multicopies and repeated sequences, the A2-A2rel gene cluster loci are not properly assembled in published *L. donovani* and *L. infantum* genomes (TriTrypDB). (B) The double- and triple-gRNA expression vectors used to target A2 and *LdMT* genes. rRNAP, *L. donovani* rRNA promoter; H, HDV ribozyme; HH, Hammerhead ribozyme. Black boxes represent the 92-bp pyrimidine track. The drawing is not to scale. (C) Western blot analysis of A2 proteins in *L. donovani* transfected with the double- or triple-gRNA expression vectors (as in panel B), with or without miltefosine selection. Equal loading of cell lysates was verified by reprobing the membrane with anti-HSP83 antibodies. (D) PCR verification of A2 null mutants using A2-specific primers L and R. The genomic DNA quality for each sample was verified by PCR with *RAD51*-specific primers.

One of the key advantages of CRISPR-Cas9 technology is that the Cas9/gRNA complex will continually scan the genome and generate double-strand DNA breaks until all the targeting sites in the genome have been deleted or mutated. This has been used successfully to inactivate multicopy family genes and endogenous retroviruses in other cell types (9, 10). A2 is a multicopy gene family and an important virulence factor for visceral *Leishmania* infection (6, 8, 11–13). There are at least 11 copies of the A2 gene of different sizes in *L. donovani* 1SC12D (8, 12) (Fig. 2A). Due to these multiple copies that also alternate with another gene termed A2rel, it was not possible to delete this gene family from *L. donovani* using the conventional gene-targeting approach (8). We therefore attempted to delete all copies of this multigene family from chromosome 22 of *L. donovani* by using the CRISPR-Cas9 method. A2 gene-coding sequences are mainly composed of 30-nucleotide repeat sequences encoding 10 amino acid repeats (11, 14) (Fig. 2A). To avoid these repeated sequences from being used as an HDR template, two gRNAs targeting the unique sequences (one targeting near the 5′ end of the A2 coding sequence and the other targeting the 3′ untranslated region) (Fig. 2A) in each of the A2 genes were designed and cloned into the dual gRNA expression vector (Fig. 2B; Data Set S2). In addition to targeting A2 genes, we also investigated whether it was possible to increase the efficiency of A2 gene deletion by coselecting for cells with CRISPR-Cas9 activity. Coselection for CRISPR-Cas9 activity was performed by targeting the *L. donovani* miltefosine transporter gene (*LdMT*) and selecting for miltefosine resistance at the same time as targeting the A2 genes. The rationale was that if one gRNA were used to target the *LdMT* gene and the other gRNA(s) targeted a different gene of interest (in this case, A2), following selection for miltefosine resistance the A2 gene would be targeted with a higher frequency. Thus, the *LdMT* gRNAa coding sequence was added into the gRNA A2a+b construct to generate a triple gRNA expression vector to determine whether A2 genes could be more efficiently deleted in these miltefosine-resistant cells (Fig. 2B).

10.1128/mSphere.00340-16.2 DATA SET S2  The representative *L. donovani* A2 gene (LinJ.22.0670; LdBPK_220670.1) sequence and the sequences of gRNA guides (green) and primers used to generate double A2 gRNA expression vectors and detect A2 deletion mutants. The locations and directions of the gRNA guide and primers in the A2 gene are indicated and underlined with an arrow. Note that because of the presence of multicopies and repeated sequences, the A2-A2rel gene cluster loci are not properly assembled in published *L. donovani* and *L. infantum* genomes. Download Data Set S2, DOCX file, 0.02 MB. Copyright © 2017 Zhang et al. 2017 Zhang et al. This content is distributed under the terms of the Creative Commons Attribution 4.0 International license.

As shown by Western blotting analysis in Fig. 2C, with prolonged culture and without cloning, the A2 genes could be completely deleted (inactivated) with this CRISPR system (Fig. 2C, lane 5). However, coselection for CRISPR-Cas9 activity with miltefosine significantly reduced the time needed to delete all A2 genes, from 4 months with no selection (Fig. 2C, lane 5) to 6 weeks with miltefosine selection (Fig. 2C, lane 4). In contrast, in the absence of miltefosine there remained detectable but diminished A2 protein expression at 6 weeks (Fig. 2C, lanes 2 and 3). Six weeks is the minimum time to establish miltefosine-resistant cells in culture. The complete deletion of A2 genes in these *L. donovani* cells was further confirmed by PCR analysis with A2-specific primers (Fig. 2D). This demonstrated that this CRISPR system can be used to delete multicopy family genes and that coselection for CRISPR-Cas9 activity can significantly reduce the time needed to obtain these deletion mutants. This is valuable, since there are many tandem gene arrays and supernumerary chromosomes in various *Leishmania* species and isolates (3–6).

This coselection observation is similar to what was reported for *Caenorhabditis elegans* and in human cells, where the co-CRISPR strategy has greatly facilitated detection of genome-editing events; particularly, CRISPR cotargeting the hypoxanthine phosphoribosyltransferase (HPRT) gene in human cells followed by 6-thioguanine selection highly enriched the cotargeting gene edited mutants (15–17). This also agrees with the observation of a bimodal distribution of CRISPR inactivation of porcine endogenous retroviruses (PERVs), where only 10% of clones exhibited complete disruption of all 62 copies of PERV *pol* genes and the remaining clones exhibited no or a low level of editing (10).

### Chromosomal translocation by targeting two sites from different chromosomes simultaneously.

**FIG 3:**
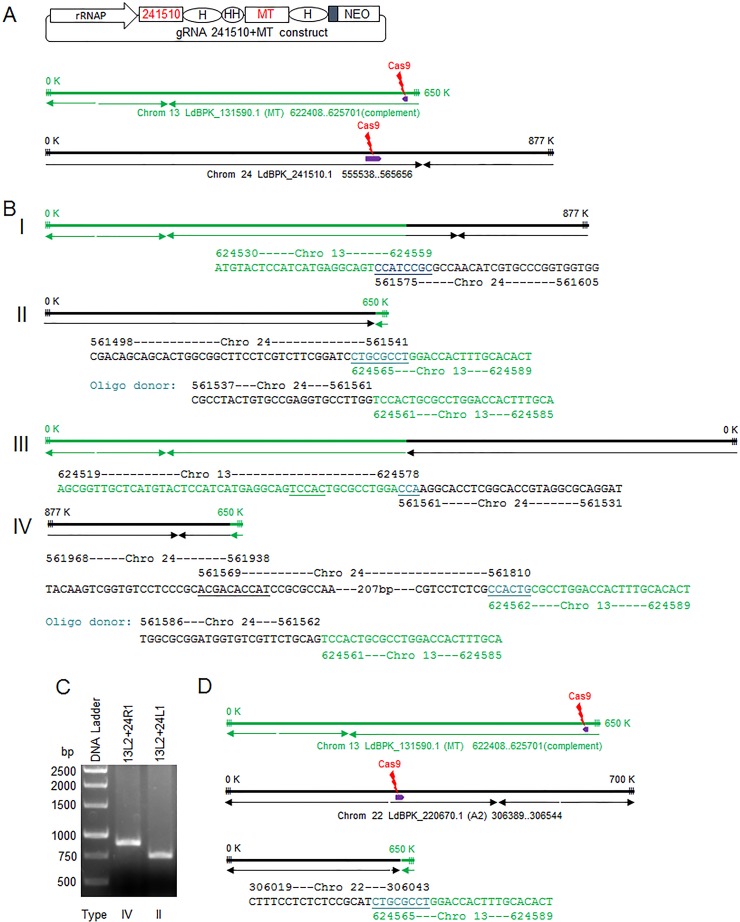
Targeted chromosomal translocations generated by targeting sites from two different chromosomes simultaneously. (A) The double-gRNA vector used to coexpress LdBPK_241510.1 targeting gRNA (241510), the *LdMT* targeting gRNAa (MT), and a schematic of chromosomes 13 and 24, with the Cas9 cleavage sites and the polycistronic transcription directions indicated. (B) Schematic of the 4 types of chromosomal translocations detected following transfection with the gRNA241510+MT coexpression vector. The chromosomal translocation junction sequences joined by MMEJ or a transfected oligonucleotide donor-directed repair are also included. Note that the polycistronic transcription directions and the numbers indicating chromosome size, which could have been altered in the newly generated fused chromosomes after translocation, are directly transferred from the parent chromosomes 13 and 24. (C) PCR detection of type II and type IV chromosomal translocations in cells expressing 241510- and MT-targeting gRNAs following transfection with the mixture of type II and type IV oligonucleotide donors (see the sequences in panel B). Primer 13L2, Ld131590L2; 24L1, Ld241510L1; 24R1, Ld241510R1. (D) Chromosomal translocation detected after *L. donovani* cells transfected with gRNA A2a+b+MT coexpression vector (Fig. 2B). For simplicity, only one A2 gene and one Cas9 cleavage site are represented for the A2-A2rel gene cluster loci in chromosome 22. See the supplemental material for all primer pairs used to detect these chromosomal translocations.

Targeted chromosomal translocations have been successfully generated in various organisms by using CRISPR-Cas9 technology, including various cancer models (18–21). The ability to generate targeted chromosomal translocations in *Leishmania* will help to investigate gene expression and identify mechanisms for the initiation and termination of polycistronic transcription and chromosome stability (22–24). To determine whether specific chromosomal translocations could be generated in *Leishmania*, a dual gRNA expression plasmid was constructed where one gRNA was targeted to the nonessential multidrug resistance gene *Ld241510* in chromosome 24 (25) (Data Set S3) and the other was targeted to the miltefosine transporter gene (*LdMT*) in chromosome 13 (7) (Fig. 3A). The hypothesis was that although most of the dsDNA breaks (DSBs) generated by Cas9 nuclease in chromosomes 13 and 24 would be repaired by intrachromosomal joining, some interchromosomal (translocation) joining could also occur. Following transfection and miltefosine selection, genomic DNA was extracted from the surviving cells, and various PCR primer pairs (one primer specific for chromosome 13 and the other for chromosome 24, each close to one of the two DSB sites) (see Data Set S3 for details), were used to investigate chromosomal translocation. As shown in Fig. 3B, it was possible to detect all four types of chromosomal translocation events resulting from the two DSBs generated simultaneously in chromosomes 13 and 24. This demonstrated that these translocations can occur regardless of the polycistronic transcription direction or the size of the new chromosome generated by translocation. In addition, following cotransfection of two oligonucleotide donors that promoted competitive translocation events (type II or type IV), both translocation PCR products were detected at similar frequencies (Fig. 3C). This further suggests that the frequency of chromosomal translocation events may not be affected by the direction of polycistronic transcription.

10.1128/mSphere.00340-16.3 DATA SET S3  The partial sequences of the LdBPK_241510.1 gene, pSPneogRNA241510+MT dual gRNA expression vector, and the sequences of the gRNA guide, oligonucleotide donors, and primers used to generate and detect translocations between chromosomes 13 and 24. The partial sequence of the pSPneogRNA241510+MT vector includes the 183-bp LdrRNAP sequence in black (its transcription initiation site [**T**] is shown in bold), the 241510 and MT gRNA coding sequences in green (the guide sequences are underlined), two 68-bp HDV ribozyme sequences in blue, and the 49-bp Hammerhead ribozyme in purple. The restriction enzymes HindIII and BamHI are highlighted in red. Download Data Set S3, DOCX file, 0.01 MB. Copyright © 2017 Zhang et al. 2017 Zhang et al. This content is distributed under the terms of the Creative Commons Attribution 4.0 International license.

It is interesting that the two gene clusters from chromosomes 13 and 24 were joined together back to back in the type I chromosomal translocation, despite transcription going in opposite directions. In contrast, the other two gene clusters were joined together head to head in the type II chromosomal translocation. Since these two gene clusters in type I chromosomal translocation lose their corresponding transcription initiation sequences, it will be interesting to see how the transcription levels of these gene clusters from the parent chromosomes are affected by these chromosomal translocations. To our knowledge, this could be the first example to show that all four types of chromosomal translocations can be generated (18–21). Interestingly, we also detected a chromosomal translocation event (Fig. 3D) in *Leishmania* cells transfected with the triple gRNA expression vector described above in Fig. 2B, in which two gRNAs were targeted to the *A2* loci in chromosome 22 and one gRNA was targeted to the *MT* locus in chromosome 13.

As expected, in the absence of oligonucleotide donors, all the chromosomal translocations were joined by MMEJ. It is interesting that two MMEJs were observed in both the detected type III and IV chromosomal translocations. In the type III chromosomal translocation detected, one MMEJ (TCCAC) joined sequences from each side of the break in chromosome 13 before the second MMEJ made the chromosomal translocation joint, which used only a 3-bp microhomology sequence (CCA). In the detected type IV chromosomal translocation, a section of chromosome 24 sequence (more than 200 bp) was reversed, which was likely caused by a flip of the single-strand DNA created by an end resection after the double-strand break and the intramolecular MMEJ (ACGACACCAT). Though the chromosomal translocation events were relatively rare and were enriched by cotargeting the *LdMT* gene in the current study, it should be feasible to isolate chromosomal translocation mutants in other specific targeting sites by using donors containing drug selection markers.

### The RNA polymerase I rRNA promoter is more efficient than the RNA polymerase III U6 promoter in driving gRNA expression in *Leishmania*.

**FIG 4:**
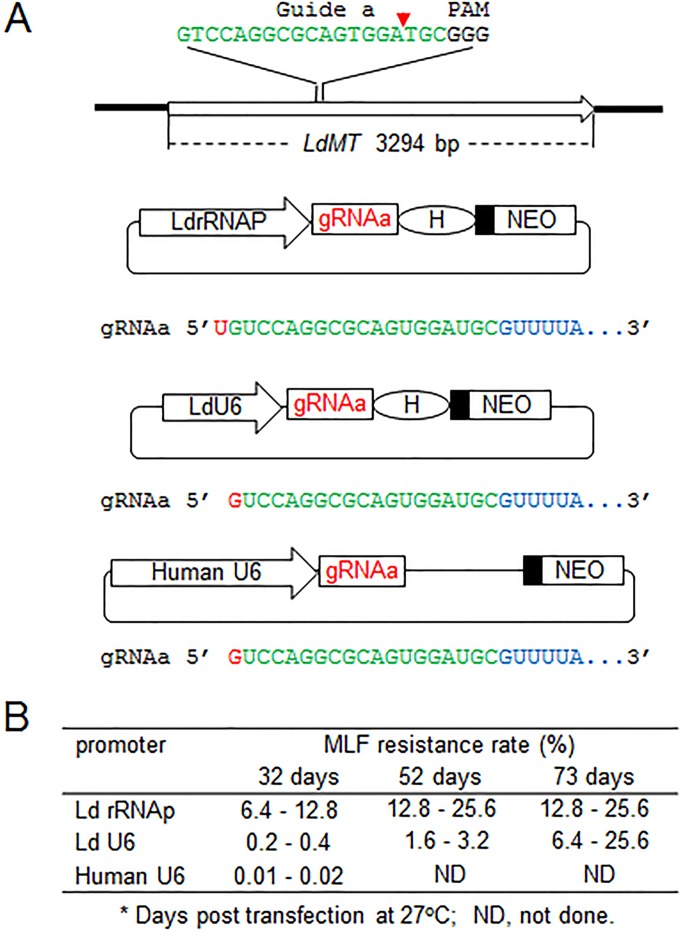
The ribosomal RNA promoter is more efficient than the U6 promoter to drive gRNA expression in *Leishmania*. (A) Different *LdMT* gRNAa expression vectors using the *L. donovani* rRNA promoter (LdrRNAP), *L. donovani* U6 promoter (LdU6), and human U6 promoter (human U6), respectively. Unlike LdrRNAP, which initiates transcription at the T residue site, *L. donovani* and human U6 promoters use a G residue to initiate transcription. As the U6 promoter terminates transcription at the 3′ end, a poly(T) (5-6) site of the gRNA coding sequence, HDV ribozyme (H) in this LdU6 promoter vector is not required, though it is able to process any of the passthrough transcripts. (B) The MLF resistance rates of *L. donovani* cells transfected with the various promoter-driving gRNAa expression vectors. The miltefosine (MLF) resistance rates were determined by limiting dilution as detailed in reference 7.

Due to its precise transcription initiation and termination, the RNA polymerase III U6 promoter has been widely used to drive gRNA expression in higher level eukaryotic cells and other protozoan parasites, including *L. major* (26–30). In contrast, the RNA polymerase I rRNA promoter used for gRNA expression has only been reported in *L. donovani* (7) and *Trypanosome cruzi* (31). We therefore compared genome-editing efficiency when gRNA expression was under control of these two different RNA polymerase promoters. An *LdMT* (*L. donovani* miltefosine transporter gene) gRNAa expression vector using the *L. donovani* U6 promoter was constructed and compared to the rRNA promoter in the pSPneogRNAaH vector, which expresses the same *LdMT* gRNAa previously described (7). In addition, we made an expression vector in which the human U6 promoter was used to direct *LdMT* gRNAa expression (Fig. 4A; Data Set S4). These* LdMT* gRNAa expression vectors were transfected into Cas9-expressing *L. donovani* promastigotes, and resistance to miltefosine was determined. As shown in Fig. 4B, the miltefosine resistance rate was much lower in the LdU6 promoter vector-transfected cells than in the rRNA promoter vector-transfected cells at 32 days posttransfection, indicating that the rRNA promoter is more efficient at driving gRNA expression in *L. donovani*. Interestingly, while the miltefosine resistance rate for cells using the rRNA promoter appeared to be stabilized at 12 to 25% in prolonged culture, the miltefosine resistance rate in cells using the LdU6 promoter was able to reach a similar level after a much longer time period of 73 days posttransfection (Fig. 4B). It is also important to note that because the rRNA promoter is a stronger promoter than the LdU6 promoter (32, 33), it was much easier to obtain G418-resistant transfectants from the rRNA promoter vector than from the LdU6 promoter vector-transfected *Leishmania* cells (data not shown). Surprisingly, the human U6 promoter was also functional in *Leishmania* and could mediate gRNA expression, though with a much lower targeting efficiency. Taken together, this comparison demonstrated that the rRNA promoter is much better than the U6 promoter in driving gRNA expression in *Leishmania*. Thus, it would be interest to explore the RNA polymerase I promoter for improving gRNA expression in other organisms, including human cells.

10.1128/mSphere.00340-16.4 DATA SET S4  Partial sequences of *LdMT* gRNAa expression vectors by using *L. donovani* rRNA promoter (LdrRNAP, pSPneogRNAaH), *L. donovani* U6 promoter (LdU6), and human U6 promoter (human U6), respectively. The promoter sequences are in black; the gRNAa coding sequences are in green; and the 68-bp HDV ribozyme coding sequences are in blue. The primers used to generate these gRNA expression vectors are also included. The restriction enzymes HindIII, BbsI, and BamHI are highlighted in red. Download Data Set S4, DOCX file, 0.01 MB. Copyright © 2017 Zhang et al. 2017 Zhang et al. This content is distributed under the terms of the Creative Commons Attribution 4.0 International license.

### Generation of gRNA and Cas9 coexpression CRISPR vectors for use with different *Leishmania* species.

**FIG 5:**
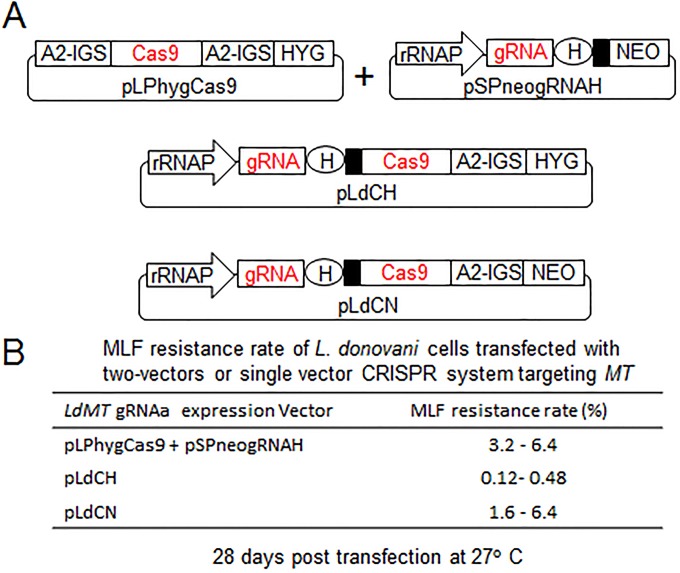
gRNA and Cas9 coexpression vectors. (A) The single vectors (pLdCH and pLdCN) derived from the previous two vectors systems. A2-IGS, A2 intergenic sequence. Please see the Fig. 2 legend for other abbreviation definitions for these vectors. The drawing is not to scale. (B) Miltefosine (MLF) resistance rates of *L. donovani* cells transfected with the two-vector or single-vector constructs expressing *LdMT* gRNAa. These are representative data of three independent experiments.

To further simplify this genome-editing system in *Leishmania*, single coexpression vectors (pLdCN and pLdCH) were constructed where the transcription of gRNA, Cas9, and a drug selection maker (neomycin or hygromycin resistance gene) were placed under control of the same rRNA promoter (Fig. 5A; Data Set S5). The *LdMT* gRNAa sequence was used to test whether these vectors could function as predicted. As shown in Fig. 5B, although there were some variations in the targeting efficiency, miltefosine-resistant cells were obtained with both pLdCNgRNAa and pLdCHgRNAa, demonstrating that the gRNA and Cas9 nuclease were properly expressed in both of these coexpression vectors. It is important to note that the *L. donovani* ribosomal promoter also functions well in other *Leishmania* species (32, 33). So far, we have successfully used the pLdCN vector to delete genes in *L. major*, *L. mexicana*, and *L. donovani* (data not shown). Therefore, a single appropriately designed gRNA construct could be used to target a conserved site (a 20-bp conserved sequence plus NGG, known as the protospacer-adjacent motif) in all three and perhaps more *Leishmania* species.

10.1128/mSphere.00340-16.5 DATA SET S5  *Leishmania* CRISPR vector pLdCN and its partial sequence. (A) Schematic of pLdCN and its guide sequence insertion site. rRNAP, *L. donovani* rRNA promoter; H, HDV ribozyme. The small filled black box represents the 92-bp pyrimidine track. The first nucleotide U of gRNA is highlighted (black), as *L. donovani* rRNAP initiates transcription at the T residue site. The drawing is not to scale. (B) The partial sequence of pLdCN (XhoI and BamHI fragment), which includes the 180-bp rRNAP sequence in black and its transcription initiation site (T) in bold, the 82-bp Cas9 binding RNA coding sequence in green, and the 68-bp HDV ribozyme coding sequence in blue. The restriction enzymes XhoI, BbsI, and BamHI are highlighted in red. Note that the guide sequence insertion site for *Leishmania* CRISPR vector pLdCH is same as that for the pLdCN vector. (C) The primers used to generate pLdCN and pLdCH. Download Data Set S5, DOCX file, 0.03 MB. Copyright © 2017 Zhang et al. 2017 Zhang et al. This content is distributed under the terms of the Creative Commons Attribution 4.0 International license.

### RAD51, a DNA recombinase involved in HDR, is essential for *L. donovani*.

**FIG 6:**
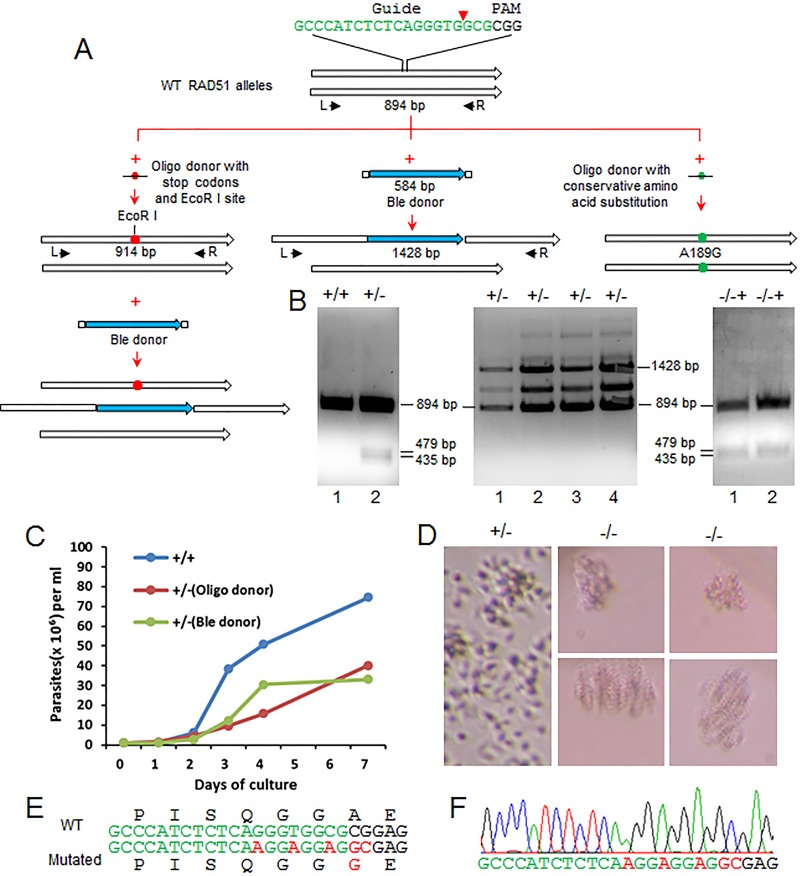
*RAD51* is essential for *L. donovani*. (A) Strategies used to generate *RAD51* disruption mutants and a mutant with a single conserved amino acid substitution. To generate various *RAD51* mutants, *L. donovani* cells were transfected with Cas9- and *RAD51*-targeting gRNA expression vectors followed by transfection of oligonucleotide donors (with stop codons and an EcoRI site or with a conservative amino acid substitution) and/or the bleomycin selection marker donor. Genomic DNA from these *L. donovani* cells (clones) were subjected to PCR, restriction enzyme digestion, and sequencing analysis. (B) PCR and restriction enzyme analysis of *RAD51* single- and double-allele disruption mutants. (Left) PCR amplification of the *RAD51* sequence with primers L and R, followed by EcoRI digestion. Lane 1, wild-type *L. donovani*; lane 2, *RAD51*^+/−^ mutant with a single *RAD51* allele disrupted by the oligonucleotide donor containing stop codons and an EcoRI site. Note that although the EcoRI-digested bands (479 and 435 bp) were detected, the 894-bp wild-type* RAD51* allele band remained in this single *RAD51* disruption mutant. (Middle) PCR analysis of phleomycin resistance clones (*RAD51*^+/−^ mutants) with primers L and R. Both the 1,428-bp bleomycin marker insertion band and the 894-bp wild-type *RAD51* allele band were detected in all these phleomycin resistance clones. Note that sequencing indicates that the additional bands detected are rearrangements of the 1,428-bp *ble* insertion bands. (Right) PCR and EcoRI digestion analysis of *Rad51*^−/−+^ mutants with one allele disrupted with stop codons and an EcoRI site containing oligonucleotide donor and the other allele with a bleomycin selection marker donor. PCR bands (not shown) similar to those in the middle panel, including the 1,428-bp *ble* insertion bands and the approximately 900-bp bands, were obtained from these −/−+ mutants. The approximate 900-bp bands were then extracted from the gel and subjected to complete EcoRI digestion. Note that the 894-bp wild-type (WT) *RAD51* allele band remained in these −/−+ mutants. These are representative data of more than 100 clones analyzed. (C) Growth curves of *L. donovani* cells targeted by *RAD51* gRNA: RAD51^+/−^ (Oligo donor), RAD51^+/−^ mutant with the oligonucleotide donor (stop codons) insertion, RAD51^+/−^ Ble donor, RAD51^+/−^ mutant with bleomycin selection marker donor insertion, and RAD51^+/+^, wild-type *L. donovani* cells expressing a control gRNAa targeting the *LdMT* gene. The data are representative of three independent experiments. (D) Microscope images showing that disruption of all *RAD51* alleles is lethal for *L. donovani*. The RAD51^+/−^ mutant cells, which continue expressing *RAD51*-targeting gRNA were cloned in 96-well plates, and cell growth was monitored by microscopy. The image for RAD51^+/−^ cells was taken 1 week after cloning; the images for Rad51^−/−^ cells were taken 3 weeks after cloning. (E) Partial sequence of the oligonucleotide donor with mutations resulting in a single conservative amino acid substitution of the RAD51 protein (A189G) and inactivation of the *RAD51* gRNA-targeting site. (F) Direct sequencing of the PCR product amplified from an *L. donovani* clone, showing both alleles of *RAD51* have been mutated to the sequence of the oligonucleotide donor (see panels A and E).

**FIG 7:**
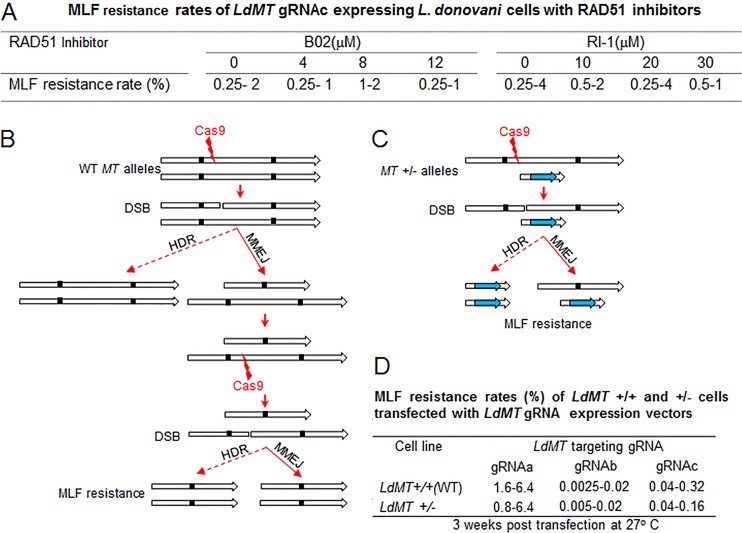
MMEJ plays a dominant role in double-strand DNA break repair in *L. donovani*. (A) RAD51 inhibitors B02 and RI-1 did not improve CRISPR gene inactivation efficiency in *Leishmania*. *LdMT* gRNAc-expressing *L. donovani* cells 6 weeks post-pSPneogRNAcH transfection were subjected to various concentrations of RAD51 inhibitors in culture medium for 2 weeks before measuring the miltefosine (MLF) resistance rate by limiting dilution. (B) Schematic showing that at least two Cas9 cleavages followed by two MMEJs or one MMEJ plus one HDR are required for wild-type (WT) *L. donovani* (MT^+/+^) to be MLF resistant (both *MT* alleles must be mutated). The small, filled black squares represent the microhomology sequences. (C) Schematic showing that only one Cas9 cleavage plus one MMEJ or one HDR are required for the *L. donovani* MT^+/−^ mutant to be MLF resistant (only the remaining WT *MT* allele needs to be mutated). This *Ld MT*^+/−^ mutant was first generated by inserting a bleomycin resistance gene into one of the *LdMT* alleles by the traditional homologous recombination method. (D) MLF resistance rates of *LdMT*^+/+^ and *Ld MT*^+/−^ cells transfected with *LdMT* gRNA -a, -b, or -c expression vectors. The data show the ranges of results from three independent experiments.

We previously observed that DSBs created by CRISPR-Cas9 in *Leishmania* were repaired by interallelic HDR, which results in error-free repair, or by MMEJ, which results in deletion mutations (7) (see Fig. 7B, below). RAD51 is a DNA recombinase required for HDR during DSB repair and is not involved in MMEJ (34–37). To determine whether inhibition of the HDR pathway would increase the frequency of MMEJ-mediated DSB repair to improve CRISPR-Cas9-directed gene inactivation efficiency, we attempted to use this CRISPR system to disrupt the *L. donovani RAD51* gene. As shown in Fig. 6A (see also Data Set S6), a plasmid carrying an *L. donovani RAD51*-specific gRNA was transfected into Cas9-expressing *L. donovani* cells, and subsequently a single-strand oligonucleotide donor with stop codons or a bleomycin resistance marker donor PCR product was cotransfected into these cells as described for the experiments shown in Fig. 1.

10.1128/mSphere.00340-16.6 DATA SET S6  *L. donovani RAD51* gene (LdBPK_280580.1) sequence and the sequences of gRNA guide, oligonucleotide donors, and primers used to generate and detect *RAD51* mutants. The locations and directions of the gRNA guide and primers in the *RAD51* gene are indicated and underlined with an arrow. Note that since the antisense oligonucleotide donor may hybridize to the corresponding gene transcripts (mRNA), which would prevent it from being used as an efficient template for oligonucleotide donor-directed repair, we were not able to generate the single-amino-acid substitution mutant with the antisense oligonucleotide donor Ld280580donor2 before using the sense oligonucleotide donor, Ld280580donor2+. Download Data Set S6, DOCX file, 0.02 MB. Copyright © 2017 Zhang et al. 2017 Zhang et al. This content is distributed under the terms of the Creative Commons Attribution 4.0 International license.

Although it was possible to disrupt one of the *L. donovani RAD51* alleles by using the donors with or without phleomycin selection, as verified by PCR analysis after cloning, one wild-type *RAD51* allele remained in the surviving cells (Fig. 6A and B). Even after two *RAD51* alleles were disrupted when we used a combination of the stop codon oligonucleotide donor and the bleomycin marker gene donor, a third wild-type *RAD51* allele still persisted (Fig. 6A and B). The *RAD51* gRNA-expressing cells did however proliferate slower than control gRNA-expressing cells, likely because the *RAD51* gRNA and Cas9 complex were continually targeting the remaining *RAD51* allele (Fig. 6C). Indeed, after cloning the *RAD51* gRNA-expressing cells (*Ld* RAD51^+^/^−^) in 96-well plates, at least 10 of these single-cell clones died out after continuous culture for 2 weeks. Interestingly, after cloning, many of these putative *RAD51* null mutants were able to continue multiplying slowly, as a clump to as many as 100 parasites before crashing, indicating it would take some time to dilute and degrade the remaining wild-type *RAD51* mRNA and proteins in these null mutant cells (Fig. 6D). Taken together, this demonstrated that like in mammalian cells, the *RAD51* gene is essential for *L. donovani*.

In contrast to *L. donovani*, a *RAD51* gene null mutant has been generated in *L. infantum* (37). Since a low frequency of homologous recombination events could still be detected in *L. infantum RAD51* null mutants, *L. infantum* may have developed ways (other recombinases) to compensate for RAD51 deficiency (37). Interestingly, while it was possible to generate *RAD51* null mutants in *Trypanosoma brucei* and *L. infantum*, RAD51 could also be essential for *L. major* and *T. cruzi*, as no *RAD51* null mutants have been reported in these latter parasites despite attempts (38-41).

Although it was not possible to disrupt or introduce stop codons into both wild-type *RAD51* alleles in surviving *L. donovani* cells, we were able to edit both *RAD51* alleles with an oligonucleotide donor designed to generate a single conserved amino acid substitution (A189G) (Fig. 6A, E, and F). This may be the first example of engineering a chromosomal single amino acid change in *Leishmania*, further revealing the importance and versatility of CRISPR-Cas9 gene editing for *Leishmania*.

### Microhomology-mediated end joining plays a dominant role in double-strand DNA break repair in *Leishmania*.

Since we were not able to generate a *RAD51* null mutant, as it is essential for *L. donovani*, we attempted to inhibit RAD51 activity by using the RAD51 inhibitors B02 and RI-1 (42–44). We reasoned that impairing HDR with these inhibitors could induce higher levels of MMEJ and increase CRISPR-Cas9 gene inactivation efficiency. However, there was no increase in *LdMT* gene inactivation through MMEJ in *LdMT* gRNAc-expressing cells after we used various concentrations of these RAD51 inhibitors (Fig. 7A), suggesting that HDR may play a less dominant role than previously anticipated for DSB repair in *Leishmania* (7). The B02 and RI-1 inhibitors did, however, confirm that RAD51 is essential, since *L. donovani* cells could not survive in medium containing more than 15 µM B02 or 70 µM RI-1, though the general toxicity of these RAD51 inhibitors might also have contributed in part to the death of these *Leishmania* cells.

The role that HDR and MMEJ play in DSB repair was further investigated by comparing the repair rates of *MT* genes in wild-type *L. donovani* (LdMT^+/+^) cells and *L. donovani* cells with one *MT* gene replaced with a bleomycin gene (LdMT^+/−^) by using traditional gene replacement. LdMT^+/+^ and LdMT^+/−^ mutants were then transfected with vectors expressing Cas9 and three different *LdMT* gene-targeting gRNAs designated a, b, and c (7). If HDR indeed played a major role in DSB repair, the miltefosine resistance rate (i.e., mutation rate) would be expected to be much higher in LdMT^+/−^ cells than in wild-type LdMT^+/+^ cells. This is because there is no wild-type *MT* allele available as an HDR template, and cleavage of the single wild-type *MT* allele would result in miltefosine resistance either through HDR or through MMEJ (Fig. 7C). In the wild-type LdMT^+/+^ cells, the majority of the DSBs generated in one of the *MT* alleles would be repaired by HDR (error free) with the remaining intact wild-type allele as the template, and it would therefore take longer for resistance to develop (Fig. 7B). Interestingly, however, there was no increase in miltefosine resistance rates in LdMT^+/−^ cells compared to the wild-type *LdMT*^*+/+*^ cells (Fig. 7D). This strongly suggests that MMEJ is more efficient than HDR in DSB repair in *Leishmania*, which agrees with the above RAD51 inhibition data.

Given that significant increases in gene-editing efficiency have been observed when using oligonucleotide and double-stranded DNA donors with only 25 nt (bp) microhomology flanking sequences in this and our previous studies (7), these donors could actually be the ideal templates for MMEJ rather than the HDR templates (45, 46). This interesting alternative is important, as we and authors of study reports on other organisms have previously attributed HDR as the sole mechanism of oligonucleotide donor-directed DSB repair (7, 47, 48). This would not, however, explain why the double-stranded donors with long homology arms (100 to 1,000 bp) were often less efficient in DSB repair than the short single-strand oligonucleotide donors (47, 48). These results also argue that Cas9 cleavage is quite efficient; it can quickly cleave the second allele following the first allele cleavage or it can generate DSBs on both alleles simultaneously.

In summary, we have described the optimization and simplification of CRISPR-Cas9 genome editing in *Leishmania*. Sequential transfections of oligonucleotide donors and cotargeting *MT* significantly increase gene-editing efficiency. The single gRNA and Cas9 coexpression vectors that were successfully used in all three tested species further simplify this CRISPR-Cas9 system for *Leishmania*. With this optimization, it was possible to delete the multicopy A2 gene family and to generate targeted chromosomal translocations, which will further advance studies on pathogenesis and polycistronic transcription control in *Leishmania*. By directly observing the death of CRISPR-targeted *RAD51* null mutant clones, we demonstrated that the RAD51 DNA recombinase is essential for *L. donovani*, confirming that this CRISPR system can be effectively used to determine the essentiality of a *Leishmania* gene. We have presented evidence to argue that MMEJ plays a more important role than HDR in DSB repair in* L. donovani*. The CRISPR-Cas9 technology described within has greatly improved the ability to manipulate the* Leishmania* genome and provides new knowledge about DNA repair in *L. donovani*. We foresee that CRISPR-Cas9 will soon be widely used in *Leishmania* research and will eventually help control and eliminate leishmaniasis.

## MATERIALS AND METHODS

### *Leishmania* strains and culture medium.

*L. donovani* 1 S/Cl2D, *L. major* Friedlin v9, and *L. mexicana* (MNYC/BZ/62/M379) used in this study were routinely cultured at 27°C in M199 medium (pH 7.4) supplemented with 10% heat-inactivated fetal bovine serum, 40 mM HEPES (pH 7.4), 0.1 mM adenine, 5 mg liter^−1 ^hemin, 1 mg liter^−1^ biotin, 1 mg liter^−1^ biopterine, 50 U ml^−1^ penicillin, and 50 µg ml^−1^ streptomycin. Cultures were passaged to fresh medium at a 20-fold dilution once a week. *Leishmania* cells after transfection with the CRISPR vector and donor DNA were sometimes cultured at 33°C or 37°C for 2 to 3 days to improve gene-editing efficiency (7).

### Plasmid construction.

All primer sequences used in this study are listed in the supplemental material (Data Sets S1 to S6).

The LdU6gRNAaH plasmid was generated as follows. (i) A 184-bp PCR fragment containing the 98-bp *L. donovani* RNA polymerase III U6 promoter was amplified from *L. donovani* genomic DNA with primers LdU6pF and LdU6pR, which also contains the *LdMT* gRNAa guide coding sequence, and digested with HindIII and BbsI. (ii) The fragment from step 1 was cloned into the HindIII and BbsI sites of the pSPneogRNAH plasmid (7) after removing the 180-bp *L. donovani* rRNA promoter to create the LdU6gRNAaH plasmid.

The humanU6gRNAa plasmid was generated as follows. (i) A 740-bp PCR fragment containing the human U6 promoter and gRNA coding sequence was amplified from plasmid pX330 (26) with primers pX330gRNAF1 and pX330gRNAR. (ii) The fragment from step 1 was digested and cloned into the HindIII and BamHI sites of pSPneo vector to create HumanU6gRNA plasmid. (iii) The *LdMT* gRNAa guide coding sequence was then inserted into HumanU6gRNA plasmid after BbsI digestion.

The pLdCH plasmid was generated as follows. (i) A 495-bp PCR fragment containing the 180-bp *L. donovani* rRNA promoter, the gRNA and HDV ribozyme coding sequences, and the 92-bp pyrimidine track was obtained from the pSPneogRNAH plasmid (7) with primers LdrRNApNde1 and pSPneoRHind3. (ii) The PCR fragment from step 1 was cloned into NdeI and HindIII sites of the pSP72 vector. (iii) The 6,595-bp HindIII and BglII fragment containing the Cas9 and hygromycin resistance genes from pLPhygCas9 (7) was subsequently cloned into the corresponding sites of the pSP72 vector in step ii to generate the pLdCH plasmid.

The pLdCN plasmid was generated as follows. (i) A 495-bp PCR fragment containing the 180-bp *L. donovani* rRNA promoter, the gRNA and HDV ribozyme coding sequences, and the 92-bp pyrimidine track was obtained from the pSPneogRNAH plasmid with primers LdrRNApXho1 and pSPneoRHind3. (ii) The PCR fragment from step i was cloned into the XhoI and HindIII sites of pSP72 vector. (iii) The 4.4-kb CAS9 fragment from the pLPhygCas9 plasmid was cloned into the HindIII and BamHI sites of the pLPneo vector (49) to generate pLPneoCas9. (iv) The 6.4-kb HindIII and BglII fragment containing the Cas9 and neomycin resistance genes from the pLPneoCas9 plasmid was subsequently cloned into the corresponding sites of the pSP72 vector in step ii to generate the pLdCN plasmid.

The gRNA 241510+*MT*(gRNAa) coexpression vector was generated by inserting the 360-bp HindIII and BamHI fragment, which contains the LdrRNAP and gRNA 241510 coding sequence from the pSPneogRNA241510H plasmid (7), into the HindIII and BglII sites of the pSPneoHHgRNAaH plasmid (7) after removing the 180-bp LdrRNAP sequence.

The gRNA A2a+b coexpression vector was generated as follows. (i) A 276-bp PCR fragment containing gRNAA2a, HDV, and hammerhead ribozymes and the gRNAA2b guide coding sequences was amplified with primers Ld220670a and Ld220670b from the gRNA 241510+*MT* coexpression vector. (ii) The PCR product from step i was digested with BbsI and inserted into the BbsI-digested pSPneogRNAH vector.

The gRNA A2a+b+*MT*(gRNAa) triple expression vector was generated by inserting the 579-bp HindIII and BamHI fragment which contained LdrRNAP, gRNA A2a+b, and ribozyme coding sequences from the gRNA A2a+b coexpression vector into the HindIII and BglII sites of pSPneoHHgRNAaH after removing the 180-bp LdrRNAP sequence.

### gRNA and primer design and synthesis.

Since current gRNA design tools developed from data for higher-order eukaryotic cells are not necessarily suitable for *Leishmania* (7), we selected gRNA guide sequences based on the relatively high activity scores in all of the following three design programs and no off-target site. The Eukaryotic Pathogen CRISPR guide RNA design tool (EuPaGDT) (http://grna.ctegd.uga.edu/) provides useful information on off-target sites and microhomology sequences flanking the DSB (9). The microhomology sequences are required for MMEJ but should be avoided if a donor will be used. Sequence scan for CRISPR (SSC) (based on human and mouse data; http://crispr.dfci.harvard.edu/SSC/) and CRISPRscan (based on zebrafish data; http://www.crisprscan.org/?page=sequence) are helpful for predicting gRNA activity based on the guide sequence alone (50, 51).

The primers for PCR were selected manually or via Primer3 (http://bioinfo.ut.ee/primer3/). All primers and oligonucleotides used in this study were ordered from Alpha DNA (Montreal, Canada).

### Other experimental procedures.

The following experimental procedures were performed as previously described: single gRNA guide sequence cloning into various gRNA expression vectors (7); *Leishmania* transfection and a limiting dilution assay to determine miltefosine resistance rates (7); *Leishmania* genomic DNA extraction, PCR, and sequencing analysis (7); A2 Western blot analysis (14).

### RAD51 inhibition assay.

The stock solutions (10 mM) of Rad51 inhibitors B02 and RI-1(catalog numbers SML0364 and 1274; Sigma) were prepared in dimethyl sulfoxide and stored at 4°C (42–44). Immediately before use, the stock solutions were diluted with *Leishmania* culture medium to a 1 mM working concentration for RI-1 and 100 µM for B02. These inhibitors were then directly added into Cas9 and *LdMT* gRNAc-expressing *Leishmania* culture (1 × 10^6^ promastigotes per ml) to a final concentration 0 to 20 µM for B02 and 0 to 100 µM for RI-1. The proper concentrations of these inhibitors in culture were maintained by adding fresh inhibitors once every 3 days for a total 4 times in a 2-week period before measuring the miltefosine resistance rate.

### Generation of LdMT^+/−^ single-knockout cells via the traditional gene replacement method.

The specific *LdMT* bleomycin-targeting fragment was generated by overlapping PCR. (i) The 666-bp *LdMT* 5′ flanking sequence with primers Ld1315905′F and Ld1315905′R, the 534-bp bleomycin expression cassette with primers 131590BleF and 131590 BleR, and the 656-bp *LdMT* 3′ flanking sequence with primers Ld1315903′F1 and Ld1315903′R were PCR amplified separately. (ii) The three PCR fragments from step i were mixed and used as PCR template for primers Ld1315905′F and Ld1315903′R to generate the specific 1,857-bp *LdMT* bleomycin-targeting fragment. *L. donovani* promastigotes transfected with this *LdMT* bleomycin-targeting fragment were selected with 100 µg/ml phleomycin. The *LdMT*^*+/−*^ single-knockout clones were verified by PCR with primer pair Ld1315905′F1 and 131590BleR.

### Accession number(s).

The pLdCN, pLdCH, and pSPneogRNA241510+MT plasmids have been deposited in Addgene under numbers 84290, 84291, and 84292, respectively. The partial sequences for these plasmids are provided in the supplemental material (Data Sets S3 and S5).

## References

[1] Alvar J, Vélez ID, Bern C, Herrero M, Desjeux P, Cano J, Jannin J, den Boer M, WHO Leishmaniasis Control Team 2012 Leishmaniasis worldwide and global estimates of its incidence. PLoS One 7:e35671. doi:10.1371/journal.pone.0035671. 22693548 PMC3365071

[2] Ready PD 2014 Epidemiology of visceral leishmaniasis. Clin Epidemiol 6:147–154. doi:10.2147/CLEP.S44267. 24833919 PMC4014360

[3] Peacock CS, Seeger K, Harris D, Murphy L, Ruiz JC, Quail MA, Peters N, Adlem E, Tivey A, Aslett M, Kerhornou A, Ivens A, Fraser A, Rajandream MA, Carver T, Norbertczak H, Chillingworth T, Hance Z, Jagels K, Moule S, Ormond D, Rutter S, Squares R, Whitehead S, Rabbinowitsch E, Arrowsmith C, White B 2007 Comparative genomic analysis of three Leishmania species that cause diverse human disease. Nat Genet 39:839–847. doi:10.1038/ng2053. 17572675 PMC2592530

[4] Downing T, Imamura H, Decuypere S, Clark TG, Coombs GH, Cotton JA, Hilley JD, de Doncker S, Maes I, Mottram JC, Quail MA, Rijal S, Sanders M, Schönian G, Stark O, Sundar S, Vanaerschot M, Hertz-Fowler C, Dujardin JC, Berriman M 2011 Whole genome sequencing of multiple *Leishmania donovani* clinical isolates provides insights into population structure and mechanisms of drug resistance. Genome Res 21:2143–2156. doi:10.1101/gr.123430.111. 22038251 PMC3227103

[5] Rogers MB, Hilley JD, Dickens NJ, Wilkes J, Bates PA, Depledge DP, Harris D, Her Y, Herzyk P, Imamura H, Otto TD, Sanders M, Seeger K, Dujardin JC, Berriman M, Smith DF, Hertz-Fowler C, Mottram JC 2011 Chromosome and gene copy number variations allow major structural change between species and strains of *Leishmania*. Genome Res 21:2129–2142. doi:10.1101/gr.122945.111. 22038252 PMC3227102

[6] Zhang WW, Ramasamy G, McCall LI, Haydock A, Ranasinghe S, Abeygunasekara P, Sirimanna G, Wickremasinghe R, Myler P, Matlashewski G 2014 Genetic analysis of Leishmania donovani tropism using a naturally attenuated cutaneous strain. PLoS Pathog 10:e1004244. doi:10.1371/journal.ppat.1004244. 24992200 PMC4081786

[7] Zhang WW, Matlashewski G 2015 CRISPR-Cas9-mediated genome editing in Leishmania donovani. mBio 6:e00861-15. doi:10.1128/mBio.00861-15. 26199327 PMC4513079

[8] Zhang WW, Matlashewski G 2001 Characterization of the A2-A2rel gene cluster in Leishmania donovani: involvement of A2 in visceralization during infection. Mol Microbiol 39:935–948. doi:10.1046/j.1365-2958.2001.02286.x. 11251814

[9] Peng D, Kurup SP, Yao PY, Minning TA, Tarleton RL 2014 CRISPR-Cas9-mediated single-gene and gene family disruption in Trypanosoma cruzi. mBio 6:e02097-14. doi:10.1128/mBio.02097-14. 25550322 PMC4281920

[10] Yang L, Güell M, Niu D, George H, Lesha E, Grishin D, Aach J, Shrock E, Xu W, Poci J, Cortazio R, Wilkinson RA, Fishman JA, Church G 2015 Genome-wide inactivation of porcine endogenous retroviruses (PERVs). Science 350:1101–1104. doi:10.1126/science.aad1191. 26456528

[11] Charest H, Matlashewski G 1994 Developmental gene expression in Leishmania donovani: differential cloning and analysis of an amastigote-stage-specific gene. Mol Cell Biol 14:2975–2984. doi:10.1128/MCB.14.5.2975. 7545921 PMC358665

[12] Zhang WW, Matlashewski G 1997 Loss of virulence in Leishmania donovani deficient in an amastigote-specific protein, A2. Proc Natl Acad Sci U S A 94:8807–8811. doi:10.1073/pnas.94.16.8807. 9238059 PMC23140

[13] Zhang WW, Mendez S, Ghosh A, Myler P, Ivens A, Clos J, Sacks DL, Matlashewski G 2003 Comparison of the A2 gene locus in Leishmania donovani and Leishmania major and its control over cutaneous infection. J Biol Chem 278:35508–35515. doi:10.1074/jbc.M305030200. 12829719

[14] Zhang WW, Charest H, Ghedin E, Matlashewski G 1996 Identification and overexpression of the A2 amastigote-specific protein in Leishmania donovani. Mol Biochem Parasitol 78:79–90. doi:10.1016/S0166-6851(96)02612-6. 8813679

[15] Kim H, Ishidate T, Ghanta KS, Seth M, Conte D Jr., Shirayama M, Mello CC 2014 A co-CRISPR strategy for efficient genome editing in Caenorhabditis elegans. Genetics 197:1069–1080. doi:10.1534/genetics.114.166389. 24879462 PMC4125384

[16] Paix A, Wang Y, Smith HE, Lee CY, Calidas D, Lu T, Smith J, Schmidt H, Krause MW, Seydoux G 2014 Scalable and versatile genome editing using linear DNAs with microhomology to Cas9 Sites in Caenorhabditis elegans. Genetics 198:1347–1356. doi:10.1534/genetics.114.170423. 25249454 PMC4256755

[17] Liao S, Tammaro M, Yan H 2015 Enriching CRISPR-Cas9 targeted cells by co-targeting the HPRT gene. Nucleic Acids Res 43:e134. doi:10.1093/nar/gkv675. 26130722 PMC4787791

[18] Blasco RB, Karaca E, Ambrogio C, Cheong TC, Karayol E, Minero VG, Voena C, Chiarle R 2014 Simple and rapid *in vivo* generation of chromosomal rearrangements using CRISPR/Cas9 technology. Cell Rep 9:1219–1227. doi:10.1016/j.celrep.2014.10.051. 25456124

[19] Maddalo D, Manchado E, Concepcion CP, Bonetti C, Vidigal JA, Han YC, Ogrodowski P, Crippa A, Rekhtman N, de Stanchina E, Lowe SW, Ventura A 2014 In vivo engineering of oncogenic chromosomal rearrangements with the CRISPR/Cas9 system. Nature 516:423–427. doi:10.1038/nature13902. 25337876 PMC4270925

[20] Chen X, Li M, Feng X, Guang S 2015 Targeted chromosomal translocations and essential gene knockout using CRISPR/Cas9 technology in Caenorhabditis elegans. Genetics 201:1295–1306. doi:10.1534/genetics.115.181883. 26482793 PMC4676527

[21] Jiang J, Zhang L, Zhou X, Chen X, Huang G, Li F, Wang R, Wu N, Yan Y, Tong C, Srivastava S, Wang Y, Liu H, Ying QL 2016 Induction of site-specific chromosomal translocations in embryonic stem cells by CRISPR/Cas9. Sci Rep 6:21918. doi:10.1038/srep21918. 26898344 PMC4761995

[22] van Luenen HG, Farris C, Jan S, Genest PA, Tripathi P, Velds A, Kerkhoven RM, Nieuwland M, Haydock A, Ramasamy G, Vainio S, Heidebrecht T, Perrakis A, Pagie L, van Steensel B, Myler PJ, Borst P 2012 Glucosylated hydroxymethyluracil, DNA base J, prevents transcriptional readthrough in Leishmania. Cell 150:909–921. doi:10.1016/j.cell.2012.07.030. 22939620 PMC3684241

[23] Reynolds D, Cliffe L, Förstner KU, Hon CC, Siegel TN, Sabatini R 2014 Regulation of transcription termination by glucosylated hydroxymethyluracil, base J, in Leishmania major and Trypanosoma brucei. Nucleic Acids Res 42:9717–9729. doi:10.1093/nar/gku714. 25104019 PMC4150806

[24] Reynolds DL, Hofmeister BT, Cliffe L, Siegel TN, Anderson BA, Beverley SM, Schmitz RJ, Sabatini R 2016 Base J represses genes at the end of polycistronic gene clusters in Leishmania major by promoting RNAP II termination. Mol Microbiol 101:559–574. doi:10.1111/mmi.13408. 27125778 PMC5038137

[25] Zhang WW, Matlashewski G 2012 Deletion of an ATP-binding cassette protein subfamily C transporter in Leishmania donovani results in increased virulence. Mol Biochem Parasitol 185:165–169. doi:10.1016/j.molbiopara.2012.07.006. 22841683

[26] Cong L, Ran FA, Cox D, Lin S, Barretto R, Habib N, Hsu PD, Wu X, Jiang W, Marraffini LA, Zhang F 2013 Multiplex genome engineering using CRISPR/Cas systems. Science 339:819–823. doi:10.1126/science.1231143. 23287718 PMC3795411

[27] Shen B, Brown KM, Lee TD, Sibley LD 2014 Efficient gene disruption in diverse strains of Toxoplasma gondii using CRISPR/CAS9. mBio 5:e01114-14. doi:10.1128/mBio.01114-14. 24825012 PMC4030483

[28] Ghorbal M, Gorman M, Macpherson CR, Martins RM, Scherf A, Lopez-Rubio JJ 2014 Genome editing in the human malaria parasite Plasmodium falciparum using the CRISPR-Cas9 system. Nat Biotechnol 32:819–821. doi:10.1038/nbt.2925. 24880488

[29] Wagner JC, Platt RJ, Goldfless SJ, Zhang F, Niles JC 2014 Efficient CRISPR-Cas9-mediated genome editing in Plasmodium falciparum. Nat Methods 11:915–918. doi:10.1038/nmeth.3063. 25108687 PMC4199390

[30] Sollelis L, Ghorbal M, MacPherson CR, Martins RM, Kuk N, Crobu L, Bastien P, Scherf A, Lopez-Rubio JJ, Sterkers Y 2015 First efficient CRISPR-Cas9-mediated genome editing in Leishmania parasites. Cell Microbiol 17:1405–1412. doi:10.1111/cmi.12456. 25939677

[31] Lander N, Li ZH, Niyogi S, Docampo R 2015 CRISPR/Cas9-Induced disruption of paraflagellar rod protein 1 and 2 genes in Trypanosoma cruzi reveals their role in flagellar attachment. mBio 6:e01012-15. doi:10.1128/mBio.01012-15. 26199333 PMC4513075

[32] Yan S, Lodes MJ, Fox M, Myler PJ, Stuart K 1999 Characterization of the Leishmania donovani ribosomal RNA promoter. Mol Biochem Parasitol 103:197–210. doi:10.1016/S0166-6851(99)00126-7. 10551363

[33] Martínez-Calvillo S, Sunkin SM, Yan S, Fox M, Stuart K, Myler PJ 2001 Genomic organization and functional characterization of the Leishmania major Friedlin ribosomal RNA gene locus. Mol Biochem Parasitol 116:147–157. doi:10.1016/S0166-6851(01)00310-3. 11522348

[34] Glover L, McCulloch R, Horn D 2008 Sequence homology and microhomology dominate chromosomal double-strand break repair in African trypanosomes. Nucleic Acids Res 36:2608–2618. doi:10.1093/nar/gkn104. 18334531 PMC2377438

[35] Glover L, Jun J, Horn D 2011 Microhomology-mediated deletion and gene conversion in African trypanosomes. Nucleic Acids Res 39:1372–1380. doi:10.1093/nar/gkq981. 20965968 PMC3045614

[36] Decottignies A 2013 Alternative end-joining mechanisms: a historical perspective. Front Genet 4:48. doi:10.3389/fgene.2013.00048. 23565119 PMC3613618

[37] Ubeda JM, Raymond F, Mukherjee A, Plourde M, Gingras H, Roy G, Lapointe A, Leprohon P, Papadopoulou B, Corbeil J, Ouellette M 2014 Genome-wide stochastic adaptive DNA amplification at direct and inverted DNA repeats in the parasite Leishmania. PLoS Biol 12:e1001868. doi:10.1371/journal.pbio.1001868. 24844805 PMC4028189

[38] McCulloch R, Barry JD 1999 A role for RAD51 and homologous recombination in Trypanosoma brucei antigenic variation. Genes Dev 13:2875–2888. doi:10.1101/gad.13.21.2875. 10557214 PMC317127

[39] McKean PG, Keen JK, Smith DF, Benson FE 2001 Identification and characterisation of a RAD51 gene from Leishmania major. Mol Biochem Parasitol 115:209–216. doi:10.1016/S0166-6851(01)00288-2. 11420107

[40] Regis-da-Silva CG, Freitas JM, Passos-Silva DG, Furtado C, Augusto-Pinto L, Pereira MT, DaRocha WD, Franco GR, Macedo AM, Hoffmann JS, Cazaux C, Pena SD, Teixeira SM, Machado CR 2006 Characterization of the Trypanosoma cruzi Rad51 gene and its role in recombination events associated with the parasite resistance to ionizing radiation. Mol Biochem Parasitol 149:191–200. doi:10.1016/j.molbiopara.2006.05.012. 16828179

[41] Passos-Silva DG, Rajão MA, Nascimento de Aguiar PH, Vieira-da-Rocha JP, Machado CR, Furtado C 2010 Overview of DNA repair in Trypanosoma cruzi, Trypanosoma brucei, and Leishmania major. J Nucleic Acids 2010:840768 doi:10.4061/2010/840768. PMC2952945 20976268

[42] Budke B, Logan HL, Kalin JH, Zelivianskaia AS, Cameron McGuire W, Miller LL, Stark JM, Kozikowski AP, Bishop DK, Connell PP 2012 RI-1: a chemical inhibitor of RAD51 that disrupts homologous recombination in human cells. Nucleic Acids Res 40:7347–7357. doi:10.1093/nar/gks353. 22573178 PMC3424541

[43] Huang F, Mazina OM, Zentner IJ, Cocklin S, Mazin AV 2012 Inhibition of homologous recombination in human cells by targeting RAD51 recombinase. J Med Chem 55:3011–3020. doi:10.1021/jm201173g. 22380680

[44] Huang F, Mazin AV 2014 A small molecule inhibitor of human RAD51 potentiates breast cancer cell killing by therapeutic agents in mouse xenografts. PLoS One 9:e100993. doi:10.1371/journal.pone.0100993. 24971740 PMC4074124

[45] Nakade S, Tsubota T, Sakane Y, Kume S, Sakamoto N, Obara M, Daimon T, Sezutsu H, Yamamoto T, Sakuma T, Suzuki KT 2014 Microhomology-mediated end-joining-dependent integration of donor DNA in cells and animals using TALENs and CRISPR/Cas9. Nat Commun 5:5560. doi:10.1038/ncomms6560. 25410609 PMC4263139

[46] Sakuma T, Nakade S, Sakane Y, Suzuki KT, Yamamoto T 2016 MMEJ-assisted gene knock-in using TALENs and CRISPR-Cas9 with the PITCh systems. Nat Protoc 11:118–133. doi:10.1038/nprot.2015.140. 26678082

[47] Böttcher R, Hollmann M, Merk K, Nitschko V, Obermaier C, Philippou-Massier J, Wieland I, Gaul U, Förstemann K 2014 Efficient chromosomal gene modification with CRISPR/cas9 and PCR-based homologous recombination donors in cultured Drosophila cells. Nucleic Acids Res 42:e89. doi:10.1093/nar/gku289. 24748663 PMC4066747

[48] Lin S, Staahl BT, Alla RK, Doudna JA 2014 Enhanced homology-directed human genome engineering by controlled timing of CRISPR/Cas9 delivery. eLife 3:e04766. doi:10.7554/eLife.04766. 25497837 PMC4383097

[49] Zhang WW, Charest H, Matlashewski G 1995 The expression of biologically active human p53 in Leishmania cells: a novel eukaryotic system to produce recombinant proteins. Nucleic Acids Res 23:4073–4080. doi:10.1093/nar/23.20.4073. 7479067 PMC307345

[50] Xu H, Xiao T, Chen CH, Li W, Meyer CA, Wu Q, Wu D, Cong L, Zhang F, Liu JS, Brown M, Liu XS 2015 Sequence determinants of improved CRISPR sgRNA design. Genome Res 25:1147–1157. doi:10.1101/gr.191452.115. 26063738 PMC4509999

[51] Moreno-Mateos MA, Vejnar CE, Beaudoin JD, Fernandez JP, Mis EK, Khokha MK, Giraldez AJ 2015 CRISPRscan: designing highly efficient sgRNAs for CRISPR-Cas9 targeting in vivo. Nat Methods 12:982–988. doi:10.1038/nmeth.3543. 26322839 PMC4589495

