# Supplementary material for: Optimized CRISPR-Cas9 Genome Editing for Leishmania and Its Use To Target a Multigene Family, Induce Chromosomal Translocation, and Study DNA Break Repair Mechanisms
Source: mSphere. 2017 Jan 18;2(1):e00340-16. doi: 10.1128/mSphere.00340-16 (PMC5244264; doi:10.1128/mSphere.00340-16)
Supplement: DATA SET S1 [file sph001172218s1.docx]

**LdBPK_131590.1 (LdMT, Miltefosine transporter):**

LdBPK_131590.1 | Leishmania donovani BPK282A1 | phospholipid-transporting ATPase 1-like protein | genomic | Ld13_v01s1 reverse | (geneStart-960 to geneEnd+480) | length=4734

-960 CACCCACGCTGCGTCGCGTTTTGCTTTATTTAGCCTCGGCTGTCTCCTCCTCTCACCTTC

-900 CTCTTCATCTGTTTTGACTGCTGTTGTTGCTGTTATCATTGCACTATTCGCCACGCCTTT

-840 TCCTCAGTTGCCTTGCCTGTCCGCCCAATTCTTTTTTATGTTTTCATCTTCTCTTCCATT 5’F1

-780 CGTTGTGGTACACTGTTGCTGTCGCTGCTGCTGACTTGGCGAGGACGCGTCTCTCTCTCT

-720 CTCTGTATTCAGGCAAGCAGTCTCTGTTCCGCCCATCCCCTTCTACCGCTCTGCCCCTTC

-660 CCCCCTCCCTCCACTACCCCTCCCCGACTCCTCAACTCCTTATATTGGTAATACTGGTCT

-600 TTTTCAATTGTTTATTTATTTGCGTGTCTTCTTCGTGTGTGTGTCGGTCGTCCTCTGCCC

-540 CCGTGAAGTCACACCCGTGCATTAGTACCCCCCCCCTACACACACGCCACACACACCACC

-480 ACGCAGGCCTTTCTGTGCGTACGACACTTTGAGCTTTTGCTTGCGTTCCTTTCCTCGCTC 5’F

-420 CACCACTCGCCTGACTGGAGCGCCACACCACGACCTGTTGCCTTCTCGTGTTGCACCGCT

-360 CACCCTTTTGGGTCGCCGTCTCCTTCCGTCATCCTATACGTGCCGCATTTCTCAGCCGTT

-300 TTCCTCTTTTCCTTCTACCCTCGCTGCAGCCCTCCCTCCGCCCTCTTTCCCTGACGATCA

-240 CACGGACACACACACATACACACACACACACACACAGTCTCTCTCTGTCTCGCGCGCGGT

-180 GTTTCTGTGTGCGTCTGACGCGGGAAGCCTTGAGCTGGCCTCCCGCTCCGTCTCCCTTCC

-120 CTCCCTTTCTTTGTTCAATGGAAAGCACCGGCCTGTCTATGCGCGTGTGTGTGTGTATGC

-60 CTGTGGTACGTCTTCGCTCTTCTTGTTCCTTTTATATACCCTCTAGGAGCTGCGGCAATC

1 ATGCCCAACCAACCGCCGTGTTGGCGCAAGTGCCTTTCCACCAGAATCTTCCCAGACAAG

61 CTCTCCAAGTCCTTCTGCTGCTTTAGCGCAGAGGCGGACGTGGACGAGGATGATGAGGTG

121 ATCGTGTACCTTAACGACCCCGAGTTGAACGCGCAGTTTAATTATCCGTCGAACTTCATT L3

181 CGTACCTCCAAGTACACACTCATCTCCTTCCTCCCACTCAGCCTCCTGTTGGAGTTCAAA 5’R

241 AAGGTGAGTAATTTGTATTTCCTCATGAACGTCATATTCAGCCTCATCCCAGGTGTGTCC

301 CCGCTAAGTCCGGCGACCTCGATTGCGCCGCTGTCCTTTGTGCTCATCGTGGCACTCATC

361 AAGGAGGGGGTGGAGGACATCAAGCGACATCAGGCCGACAACCGCGCCAACTCGATTTTA

421 GTGCAGGTACTGCGAAACGGCAAGCTCGTCTCGGTGCACAGCAAGGACATCCACCCTGGT

481 GACGTCATGCGTATCAAGAACGGCGAGGAGGTGCGCGCCGATGTCGTCATGCTCGCCTCG

541 TCCGTCGAGGAAGGACAGGCATTTATAGACACATGTAACCTGGACGGCGAGACGAACCTG

601 AAGTCGCGCAAGGCTCTGGAAGCCACCTGGGCGCTCTGCGAAGTCGAGGCAATCATGAAT

661 AGCACAGCCGTACTGCACACGAGCAAGCCAGACCCAGGGTTGCTGTCGTGGGCAGGGCTG L2

721 TTGGAAATCAATGGCGAGGAGCACGCTCTCTCGCTGAACCAGTTCCTGTATCGCGGCTGC

781 GTGTTACGCAACACGGACTGGGTGTGGGGCATGGTTGCCTACGCAGGGGTCGACACGAAG

841 CTGTTCCGAAACTTGAAGCCAAAACCGCCAAAGTCGTCGAACCTCGACCGCAAGCTGAAC L&L1

901 TACTTTATCATAGCCATCCTCATATTCCAGAACATCATGCTCTTCATCTTAGCCTCCATG

961 GCAGTGTGGTGGAACAGCAAGTACCGGGAAACGCCCTACCTCCGCTTCTTTATCAGCTTT gRNAc

1021 CGCAAGAACGTAACTCTGTGGGGATACCGCTACTTGAGTTACTTCATTTTGCTGAGCTAC gRNAb

1081 TGCGTGCCCATCTCGCTGTTCATCACGATTGAAGTGTGCAAAGTGGTCCAGGCGCAGTGG gRNAa

1141 ATGCGGGTGGACTGCCTCATGATGGAGTACATGAGCAACCGCTGGCGGCACTGCCAGCCG

1201 AACACGTCGAACCTCAACGAGCAGCTAGCAATGGTGCGCTTCATCTTCAGCGACAAAACT R2

1261 GGGACGTTGACAGAGAACGTCATGAAGTTCAAGCTAGGCGACGCTCTCGGTAATCCGATC R&R1

1321 GACGCCGACAATCTGGACGAGTGCATCGCGCAGCTGCGCAAGGAGGCCGAGTCGAAGGGG

1381 CTAGGCCCGCTGCAAGAGTACTTTCTCGCGCTGGCCCTGTGCAACACGGTTCAGCCCTTC

1441 AAGGACGACACGGATGACTTGGGTGTTGTCTACGAAGGCAGCTCCCCAGACGAGGTGGCG

1501 CTGGTCGAGACCGCTGCTGCTGTCGGCTATCGCCTCATCAGCCGTACGACAAAGTCCATC

1561 ACGCTACTCCTGCACGATGGGACGCGCAAGGTATACAACATCCTCGCCACACTGGAGTTC

1621 ACGCCGGACCGCAAGATGATGAGCATCATCGTCGAGGACAGCGACACCAAAAAAATTACG

1681 CTGTACAATAAGGGGGCCGACAGTTTCATCAGGCCGCAGCTGAGCCGCGCCCCGGATGTG

1741 CAGGGGCACATCGAAAATGTCGAGATCCCTCTGACGGAAATGTCCTCGTCGGGGCTCCGC

1801 ACGCTGCTTGTGTGCGCCAAGGATATCACACGGCGCCAGTTCGACCCATGGTTCGAGAAG

1861 TTCGTCGAAGCCGGCAAGTCCCTGCACAACCGCAGCTCCAATATTGATAAAGTCTGCTTA

1921 GAGATGGAGCAAGATATGCGGCTCGTCGGTGCCACCGCTATCGAGGACAAGCTGCAAGAC

1981 GAGGTCCCTGAGACACTGTCCTTCTTCTTGAGCGCCGGTGTAATCATTTGGATGCTCACT

2041 GGCGACAAGCGCGAGACCGCCGTGACGATCGCTGCAACGTCGACCCTGTGCGACCCGCGC

2101 AACGACTTCATCGACCACATCGACATTGGTCATCTGAATTCATCGGATCCCAAGGCGATT R3

2161 GAGCGCGTAGGGCGCGACCTCGAAGTGGTGGAGCAGCACATCGCGCTCAAGGGGACCCAC

2221 AAGGAGCGGCGCTGCACCTTGGTCATCGACGGCCCAGCGCTGAACATCGCAATGGAGCAC

2281 TACTTTGACCAGTTCCTGCGCCTCTCCCATCAGGTCAACTCCGCCGTCTGCTGTCGTCTC

2341 ACGCCGATCCAGAAGGCAACCGTCGTTCGCATGTTCCAGAAGTCAACCGGTAAGACAGCG

2401 CTGGCCATCGGTGACGGCGCCAACGACGTGTCCATGATCCGGGAGGGGCGTGTGGGCGTG

2461 GGCATTATTGGGCTGGAAGGTGCACACGCCGCCCTCGCCGCCGACTACGCGATTCCGCGG

2521 TTCAAGCACCTGCGCCGCCTATGCGCGGTGCATGGCCGCTACTCGCTCTTCCGCAACGCC

2581 AGCTGCATTCTGGTTAGCTTCCACAAGAACATTACTGTGTCGGTGGTGCAGTTCATCTTC

2641 GCCTTCTACGTCGGCTTCTCGGGGCTAACACTCTTTGATGGATGGATGCTGACCTTCTAC

2701 AACGTCCTTCTAACAAGTATCCCACCCTTCTTCATGGGTATATTCGATAAGGACCTCCCC

2761 GAAGATGCCCTGCTGGAGCGGCCGAAGCTGTACACACCGTTGTCGCATGGCGAGTACTTT

2821 AACCTGGCGACGCTTCTGCGGTGGTTCGTCGAATCACTAACAACAGCGGTGATCCTCTTC

2881 TATGCTGCTTACCCGACATTGATCCGTCAAGACGGTTCCCATCAGCGCTACACCGGCGGC 3’F

2941 GAGACCGGCACGCTCGTGTTCAGCGGCTTGATCCTCGTCATTCAAACTCGCTTCATCCTG

3001 CAGATCCGCTACTGGCAGTGGCTGCAGGTGTTTGGCATGGCGATGTCGATTTTTCTCTTT

3061 CTGTTGTTGTTTCTCGTCTACTCCGCCATTCCCTCAGTCTTCAGTGACACGAATTTCTAC

3121 TACCAAGCCTTCGATCTCATGTCGACCGCCAAGTACTGGTTTTTCCTGCTCCTCTACGTT 3’F1

3181 GGCACCGAGGTGGTGGTCGTACTCGGCGTCATGACGTTCCAGAAGAACCTCTTCCCTACC

3241 CTGCGCGACGTCGCGGAGCGACAGTACGCTGTTCAAAACGGTGGAAAGCTGTGAAGATTG

+7 TATGCCAGTGACTAGGAAGAGGATCCAAGCACTGGGTGGGGGGGCGACAATGAGGGCAAC

+67 GCGTGGCGCTGGACCGTAGGTTGGCCCGAGTGAATGTCTGTGCTTGGTGGTGTCACTTGG

+127 GGAGGGCGGAGCGTGGGCAGAGCATCGGGGAGAGAAGTGCGTGCACATGCGAGCGGCTTG

+187 GTATGTGGCTGCACTCCTGCCGCATCCTACCTCTGCGTCGCTTGTACCTCCCTCTCTGTG

+247 TCTCTGTCAGGTGCGCGTGCGCCTGCCTGGCTTTGACTTTTAGGGGCTACTCCCTCTTAT

+307 CTCCTACCTAATCCGCATCGAACATGGGTTCGTGTTTTTTTTTCGCGTTGATGTTCTCAC

+367 TTTCACATGCATGTCTGCTTGCCTTTGCGCGTGTGTGTATGTGTGTGTCTACCTCTGTGG

+427 GTGGGCATGTGTACGTGTGTGCCGGCAGCGGTTGGCTGTGGAACGTCTTTTTTT 3’R

Ld131590L 5’ cccaagcttGCTGTTCCGAAACTTGAAGC

Ld131590R 5’ ccgagatctGTCGATCGGATTACCGAGAG

Ld131590L1 5’ GCTGTTCCGAAACTTGAAGC

Ld131590R1 5’ GTCGATCGGATTACCGAGAG

Ld131590L2 5’ CAGGGCTGTTGGAAATCAAT

Ld131590R2 5’ ACCATTGCTAGCTGCTCGTT

Ld131590L3 5’ CTTAACGACCCCGAGTTGAA

Ld131590R3 5’ ATGTCGATGTGGTCGATGAA

Ld1315905’F1 5’ CTTTTCCTCAGTTGCCTTGC

Ld1315905’F 5’ CTTTTGCTTGCGTTCCTTTC

Ld1315905’Fh 5’ cccaagCTTTTGCTTGCGTTCCTTTC

Ld1315905’R 5’ TGAGTGGGAGGAAGGAGATG

Ld1315905’Rh 5’ cccaagctTGAGTGGGAGGAAGGAGATG

Ld1315903’F 5’ GCTGCTTACCCGACATTGAT

Ld1315903’R 5’ AAAGACGTTCCACAGCCAAC

Ld1315903’Rb 5’ ccgagatctAAAGACGTTCCACAGCCAAC

131590cdonor 5’ AGTACCGGGAAACGCCCTACCTCCGTGAGTAGGTAGCTTCTTTATCAGCTTTCGCAAGAAC

131590BleF 5’ CATCTCCTTCCTCCCACTCAATCTTCATCGGATCGGGTAC

131590BleR 5’ ACATGAGATCGAAGGCTTGGTCAGTCCTGCTCCTCGGCCA

Primer pairs used to detect translocations between chromosome 13 and 22:

Ld131590L2 + Ld220670L

**S. 1** *L. donovani* Miltefosine Transporter gene (*LdMT;* LdBPK_131590.1) sequence and the sequences of gRNA guides, oligonucleotide donors and primers used to generate and detect *LdMT* mutants. The locations and directions of gRNA guides and primers in *LdMT* gene are indicated and underlined with an arrow. Some of these primers had restriction enzyme site added to their 5’ end to facilitate cloning of the PCR product.
