# Supplementary material for: Optimized CRISPR-Cas9 Genome Editing for Leishmania and Its Use To Target a Multigene Family, Induce Chromosomal Translocation, and Study DNA Break Repair Mechanisms
Source: mSphere. 2017 Jan 18;2(1):e00340-16. doi: 10.1128/mSphere.00340-16 (PMC5244264; doi:10.1128/mSphere.00340-16)
Supplement: DATA SET S2 [file sph001172218s2.docx]

**A2 sequence (LinJ.22.0670):**

LinJ.22.0670 | Leishmania infantum JPCM5 | A2 protein | genomic | LinJ.22 forward | (geneStart-600 to geneEnd+976) | length=2940

CCGGGCCTTGCGGTGTGGATGCGAGCATCGCGCATTGCCGGCCACCGCCGCGTGCCAAAA

GCACACACATCGCTCCCCCTCACGTGGTGAGCGTCTGCGCGTGTGTGTCTGTGTGCATAA

ATGCGTACGTGTGCGCTCGGTCCACGGTCACCATCGCGCCCCGCGGCATCGGTGCTCGCA

CCTTTCCATTCTCTCCGTACGGCGTTCGCCACTCTGCTAACTCCTCTCCCCTTTCCTCTC

TCCGCATCTGCGCCTTGTGTGCCGTGTGTGCGCCCCACACGTACTCTACACGCCTTCTCC

GCATGCGCTCCTCGCCTGCCTGAAACGATGACGTGCGCCGCCACTCGCTCACCCGCTCCC

ATCCACATGCATTCCTGCACCCTACAGCGACTCTAAGCCGTACATTCCATCTCCTCAACA

CCCACTCGCTTCCTTCTGCTCTCACCTATTACTTCGCCAGCCCACATATCTGCTATAACC

TGCCCTCCCCCACCCGCTTCCCACACATCCGCCACCGCTACGAAGCCTCGAGCTCCCCCA

GCGACCCTCTCGGCAACGCGAGCGCCACAGTCCCCCCACGCACAACATTGACCGAGCACA

ATGAAGATCCGCAGCGTGCGTCCGCTTGTGGTGTTGCTGGTGTGCGTCGCGGCGGTGCTC

GCACTCAGCGCCTCCGCTGAGCCGCACAAGGCGGCCGTTGACGTCGGCCCGCTCTCTGTT

GACGTTGGCCCGCTCTCCGTTGGTCCGCAGTCTGTTGGCCCGCTCTCCGTTGGCCCGCAG

TCTGTTGGCCCGCTCTCTGTTGACGTTGGCCCGCTCTCCGTTGGTCCCCAGTCTGTTGGC

CCGCTCTCTGTTGACGTTGGCCCGCTCTCCGTTGGTCCGCAGTCTGTTGGCCCGCTCTCC

GTTGGCCCGCAGGCTGTTGGCCCGCTCTCCGTTGGCCCGCAGTCTGTTGGCCCGCTCTCT

GTTGACGTTGGCCCGCAGGCTGTTGGCCCGCAGTCCGTCGGCCCGCTCTCCGTTGGCCCG

CAGTCTGTTGGCCCGCTCTCCGTTGGCCCGCAGTCTGTTGGCCCGCTCTCTGTTGGCCCG

CTCTCCGTTGGTCCGCAGTCTGTTGGCTCGCTCTCCGTTGGCCCGCAGTCCGTCGGCCCG

CTCTCTGTTGGCCCGCTCTCTGTTGACGTTGGCCCGCAGGCTGTTGGCCCGCTCTCCGTC

GGTCCGCAGGCTGTTGGCCCGCTCTCCGTTGGTCCGCAGTCCGTCGGCCCGCTCTCCGTT

GGCCCGCAGTCCGTTGGCCCGCTCTCCGTTGGTCCGCAGTCTGTCGGCCCGCTCTCTGTT

GGCCCGCAGTCCGTCGGCCCGCTCTCTGTGGGCCCGCAGTCCGTTGGCCCGCTCTCCGTT

GACGTTGGTCCGCAGTCCGTTGGCCCGCTCTCCGTTGGTCCGCAGTCCGTCGGCCCGCTC

TCCGTTGGCCCGCAGTCCGTTGGCCCGCTCTCCGTTGGCCCGCAGTCTGTTGGCCCGCTC

TCCGTTGGTCCGCAGTCCGTCGGCCCGCTCTCCGTTGGCCCGCAGTCCGTTGGCCCGCTT

TCTGTTGGCCCGCAGGCTGTTGGCCCGCTCTCTGTCGACGTTGGCCCGCAGTCCGTCGGC

CCGCTCTCTGTTGGCCCGCAGGCTGTCGGCCCGCTCTCCGTTGGTCCGCAGTCCGTCGGC

CCGCTCTCCGTTGGTCCGCAGTCCGTCGGCCCTCTCTCTGTTGACGTTGGTCAGCAGTCC

GTTGGCCCGCTCTCCGTTGGCCCGCAGTCTGTTGGCCCGCTCTCTGTTGGCCCGCAGTCC

GTCGGCCCGCTCTCCGTTGGCCCGCAGGCTGTTGGCCCGCTCTCCGTTGGCCCGCAGGCT

GTTGGCCCGCTCTCCGTTGGTCCGCAGGCTGTTGGCCCGCTCTCCGTTGGTCCGCAGTCC

GTCGGCCCGCTCTCCGTTGGCCCGCAGGCTGTTGGCCCGCTCTCCGTTGGTCCGCAGGCT

GTTGGCCCGCTCTCCGTTGGTCCGCAGTCCGTTGGCCCGCTCTCGGTTGGCCTGCAGGCT

GTTGACGTTTCTCCGGTGTCTTAAGGCTCGGCGTCCGCTTTCCGGTGTGCGTAAAGTATA
TGCCATGAGGCATGGTGACGAGGCAAACCTTGTCAGCAATGTGGCATTATCGTACCCGTG
CAAGAGCAACAGCAGAGCTGAGTGTTCAGGTGGCCACAGCACCACGCTCCTGTGACACTC
CGTGGGGTGTGTGTGACCTTGGCTGCTGTTGCCAGGCGGATGAACTGCGAGGGCCACAGC
AGCGCAAGTGCCGCTTCCAACCTTGCGACTTTCACGCCACAGACGCATAGCAGCGCCCTG
CCTGTCGCGGCGCATGCGGGCAAGCCATCTAGATGCGCCTCTCCACGACATGGCCGGAGG
CGGCAGATGAAGGCAGCGACCCCTTTTCCCCGGCCACGACGCCGCGCTGAGGCGGGCCCC
ACAGCGCAGAACTGCGAGCGCGGTGCGCGGGCGCTGTGACGCACAGCCGGCACGCAGCGT
ACCGCACGCAGACAGTGCATGGGGAGGCCGGAGGAGCAAGAGCGGTGGACGGGAACGGCG
CGAAGCATGCGGCACGCCCTCGATGTGCCTGTGTGGGCTGATGAGGCGCGGATGCCGGAA
GCGTGGCGAGGGCATCCCGAGTTGCACCGTCGAGTCCTCCAGGCCCGAATGTGGCGAGCC
TGCGGGGAGCAGATTATGGGATGCGGCTGCTCGAAGCGACCGAGGGCGCTGACCGGAAGG
TGGCCCACTTCCTCCTCGGGCCTGTGCGGCATCCGCCCTCGATCGGGAGCCCGAATGGTG
GCCGCGCGGGTGAAGGCGTGCCGCCCACCCGCGTCTCCGTGTGGCGCCGCTGGGGGCAGG
TGCGCTGTGGCTGTGTATGTGCGCTGATGTGCTGACTTGTTCGTGGTGGGCTATGGGCAC

Ld220670a 5’ ATCGAAGACCTTTGTCCACAAGCGGACGCACGCTGGTTTTAGAGCTAGAAATAGCAAG

Ld220670b 5’ ATCGAAGACCCAAACCCTGGCAACAGCAGCCAAGGTCACCATGACGAGCTTACTC

Use gRNA241510+MT co-expression vector as PCR template to get

following 276 bp PCR product:

ATCGAAGACCTTTGTCCACAAGCGGACGCACGCTGGTTTTAGAGCTAGAAATAGCAAGTT

AAAATAAGGCTAGTCCGTTATCAACTTGAAAAAGTGGCACCGAGTCGGTGCTTTTTTGGC

CGGCATGGTCCCAGCCTCCTCGCTGGCGCCGGCTGGGCAACATGCTTCGGCATGGCGAAT

GGGACGGATCTCACCATCTGATGAGTCCGTGAGGACGAAACGAGTAAGCTCGTCATGGTG

ACCTTGGCTGCTGTTGCCAGGGTTTGGGTCTTCGAT

Ld220670L 5’ CTTTCCTCTCTCCGCATCTG

Ld220670R 5’ AAACGCAACACCACAAGCGGACGC

Ld220670R1 5’ CCACGAACAAGTCAGCACAT

**S. 2**  The representative *L***.** *donovani* A2 gene (LinJ.22.0670; LdBPK_220670.1) sequence and the sequences of gRNA guides (green) and primers used to generate double A2 gRNAs expression vector and detect A2 deletion mutants. The locations and directions of gRNA guide and primers in A2 gene are indicated and underlined with an arrow. Note: Because of the presence of multicopies and repeated sequences, the A2-A2rel gene cluster loci are not properly assembled in published *L. donovani* and *L. infantum* genomes.
