# Supplementary material for: Optimized CRISPR-Cas9 Genome Editing for Leishmania and Its Use To Target a Multigene Family, Induce Chromosomal Translocation, and Study DNA Break Repair Mechanisms
Source: mSphere. 2017 Jan 18;2(1):e00340-16. doi: 10.1128/mSphere.00340-16 (PMC5244264; doi:10.1128/mSphere.00340-16)
Supplement: DATA SET S3 [file sph001172218s3.docx]

**LdBPK_241510.1 (partial sequence):**

GCACTTGGCATTTTTGTGGCGTCGCCGATGGACTCCGCCGAGGCAAGGCGGTGCGTCAGC
GCGGAGCAGACGCTTTCGGCTGTACCCGGCGCTGATGCAGCAGCGGCGCAGAGGGCGCTG
GCCGGCATCGCCTCGACAGCTGCTGCCGCGGCTGCTGCGGCCAAGACTTTCATGGCAGCA
GGCGGTGCGTCGCCGGGTGGCTACGCTCGAAGCGAGGCGTCGAAGTTGTCTGCCCGTAGC
CTCGCTGACGATCGCGCCAGTGTGTGCTCGGTTACCACTAAGAACGCCTCACAGCTTAGC
CCCACCGGCCTGGAAGGCGACGACAGCAGCACTGGCGGCTTCCTCGTCTTCGGATCCTGC
GCCTACTGTGCCGAGGTGCCTTGGCTGCAGAACGACACCATCCGCGCCAACATCGTGCCC
GGTGGTGGCCCGGTGCTGGAGCGTTGGTACTGCACGGTGCTGCGTGCGTGCGCGCTGAGC
GCGGAGGTGACGGCGCTGCCGCACGGTGACGCGACCGTGATTGGCGAGAGAGGGGAGCTG
CTGTCGCTAAGCATGCGCTGCCGCATCGCCATCGCGCGGGCCGTGTACTCCAAGTCGCAC
GTGTATCTCATGGACAGCGTCCTCTCGCCACTGGAGCCTCCCATTCAGGAGCACATTATC
CGCGAGGTGTTCCACAGGCTACTGCGCAAGAAGACGATTGTTTTGGCCAGCAACGTCGGA
CTGCGATCGCTGCGGCCGCATCGCGTTTTCAGCGTCGTGAATGGCGTCGTGCGGGAGGAC
ACCGACTTGTACACGGCCGTCTCCTCTCTGCATGCCCGCGGTGACGAGGATGACGAGGAG
GCACTGAT

PstI241510+ 5’TTGTGGTGTCGTTCTGCAGCCA

PstI241510- CCACAGCAAGACGTCGGTCAAA 5’

pSPneogRNA241510+MT(Hind III and BamH I fragment):

|  | AAGCTTGTGAGTTATGAGGTCTGCGATTGACGTAGGAGTTGCAAGGGGGAGGGGGTATGA  ACGGGGTGGGTAGAGCTTTTTTAGGTGGAAGTAGTGAGAGGGTGGGCTTGAGAGAATTTG  AGGTGTGTTCGTGATGTGTGGATCTTATCGGGGGCTCGGTTGAGTTTTTGGTTTGGTGAT  TTTG**T**GGTGTCGTTCTGCAGCCAGTTTTAGAGCTAGAAATAGCAAGTTAAAATAAGGCTA GTCCGTTATCAACTTGAAAAAGTGGCACCGAGTCGGTGCTTTTTTGGCCGGCATGGTCCC AGCCTCCTCGCTGGCGCCGGCTGGGCAACATGCTTCGGCATGGCGAATGGGACGGATCTC  ACCATCTGATGAGTCCGTGAGGACGAAACGAGTAAGCTCGTCATGGTGGTCCAGGCGCAG TGGATGCGTTTTAGAGCTAGAAATAGCAAGTTAAAATAAGGCTAGTCCGTTATCAACTTG AAAAAGTGGCACCGAGTCGGTGCTTTTTTGGCCGGCATGGTCCCAGCCTCCTCGCTGGCG  CCGGCTGGGCAACATGCTTCGGCATGGCGAATGGGACGGATCC |
| --- | --- |

Ld241510L 5’GCACTTGGCATTTTTGTGG

Ld241510R 5’GAACACCTCGCGGATAATGT

Ld241510L1 5’AGACGCTTTCGGCTGTACC

Ld241510R1 5’ATCAGTGCCTCCTCGTCATC

Ld13-24S donor: 5’TGCAAAGTGGTCCAGGCGCAGTGGACTGCAGAACGACACCATCCGCGCCA
Ld13-24R donor: 5’TGCAAAGTGGTCCAGGCGCAGTGGACCAAGGCACCTCGGCACAGTAGGCG

Primer pairs used to detect translocations between Chromosomes 13 and 24:

Ld131590L2 + Ld241510L1

Ld131590L2 + Ld241510R1

Ld131590R2 + Ld241510L1

Ld131590R2 + Ld241510R1

**S. 3** The partial sequences of LdBPK_241510.1 gene, pSPneogRNA241510+MT dual gRNA expression vector and the sequences of gRNA guide, oligonucleotide donors and primers used to generate and detect translocations between Chromosomes 13 and 24. The partial sequence of pSPneogRNA241510+MT vector includes the 183 bp LdrRNAP sequence in black (its transcription initiation site **T** in bold), the 241510 and MT gRNA coding sequences in green (the guide sequences underlined), two 68 bp HDV ribozyme sequences in blue and the 49 bp Hammerhead ribozyme in purple. The restriction enzymes Hind III and BamH I are highlighted in red.
