# Supplementary material for: Optimized CRISPR-Cas9 Genome Editing for Leishmania and Its Use To Target a Multigene Family, Induce Chromosomal Translocation, and Study DNA Break Repair Mechanisms
Source: mSphere. 2017 Jan 18;2(1):e00340-16. doi: 10.1128/mSphere.00340-16 (PMC5244264; doi:10.1128/mSphere.00340-16)
Supplement: DATA SET S4 [file sph001172218s4.docx]

pSPneogRNAaH (Hind III and BamH I fragment):

AAGCTTGTGAGTTATGAGGTCTGCGATTGACGTAGGAGTTGCAAGGGGGAGGGGGTATGA
ACGGGGTGGGTAGAGCTTTTTTAGGTGGAAGTAGTGAGAGGGTGGGCTTGAGAGAATTTG
AGGTGTGTTCGTGATGTGTGGATCTTATCGGGGGCTCGGTTGAGTTTTTGGTTTGGTGAT
TTTG**T**GTCCAGGCGCAGTGGATGCGTTTTAGAGCTAGAAATAGCAAGTTAAAATAAGGCT
AGTCCGTTATCAACTTGAAAAAGTGGCACCGAGTCGGTGCTTTTTTGGCCGGCATGGTCC
CAGCCTCCTCGCTGGCGCCGGCTGGGCAACATGCTTCGGCATGGCGAATGGGACGGATCC

LdU6 gRNAaH (Hind III and BamH I fragment):

AAGCTTATCGGATTCAGAGTCCGAGGTGATAACCGCTACACTATAGGAGCACGTTGGAAA GCGCTGCAATGACGGGCCTTCACTAAATTTTGAAAACGCATCTCCACAACTCGTTGTCTG TTTCGTTTTCTTGTGATTCTCTTGAATGTCCAGGCGCAGTGGATGCGTTTTAGAGCTAGA

AATAGCAAGTTAAAATAAGGCTAGTCCGTTATCAACTTGAAAAAGTGGCACCGAGTCGGT

GCTTTTTTGGCCGGCATGGTCCCAGCCTCCTCGCTGGCGCCGGCTGGGCAACATGCTTCG

GCATGGCGAATGGGACGGATCC

LdU6pF 5’ ACCAAGCTTATCGGATTCAGAGTCCGAG

LdU6pR 5’ ATCGAAGACCCAAACGCATCCACTGCGCCTGGACATTCAAGAGAATCACAAGAAAACG

PRODUCT SIZE: 184 bp

Human U6 gRNAaH (Hind III and BamH I fragment):

AAGCTTGTTTCGCCACCTCTGACTTGAGCGTCGATTTTTGTGATGCTCGTCAGGGGGGCG GAGCCTATGGAAAAACGCCAGCAACGCGGCCTTTTTACGGTTCCTGGCCTTTTGCTGGCC

TTTTGCTCACATGTGAGGGCCTATTTCCCATGATTCCTTCATATTTGCATATACGATACA

AGGCTGTTAGAGAGATAATTGGAATTAATTTGACTGTAAACACAAAGATATTAGTACAAA

ATACGTGACGTAGAAAGTAATAATTTCTTGGGTAGTTTGCAGTTTTAAAATTATGTTTTA

AAATGGACTATCATATGCTTACCGTAACTTGAAAGTATTTCGATTTCTTGGCTTTATATA

TCTTGTGGAAAGGACGAAACACCGTCCAGGCGCAGTGGATGCGTTTTAGAGCTAGAAATA

GCAAGTTAAAATAAGGCTAGTCCGTTATCAACTTGAAAAAGTGGCACCGAGTCGGTGCTT

TTTTGTTTTAGAGCTAGAAATAGCAAGTTAAAATAAGGCTAGTCCGTTTTTAGCGCGTGC

GCCAATTCTGCAGACAAATGGCTCTAGAGGTACCCGTTACATAACTTACGGTAAATGGCC

CGCCTGGCTGACCGCCCAACGACCCCCGCCCATTGACGTCAATAGTAACGCCAATAGGGA

CTTTCCATTGACGTCAATGGGTGGAGTATTTACGGTAAACTGCCCACTTGGCAGTACATC

AAGTGTATCATATGCCAAGTACGCCCGGATCC

pX330gRNAF1 5’CCCAAGCTTGTTTCGCCACCTCTGACTTG

pX330gRNAR 5’CCGGGATCCGGGCGTACTTGGCATATGAT

pX330gRNAF1+pX330gRNAR 739 bp

Ld131590+3 5’CACCGTCCAGGCGCAGTGGATGC

Ld131590- CAGGTCCGCGTCACCTACGCAAA 5’

**S. 4** The partial sequences of *LdMT* gRNAa expression vectors by using *L. donovani* ribosomal RNA promoter (LdrRNAP, pSPneogRNAaH), *L. donovani* U6 promoter (LdU6) and Human U6 promoter (Human U6) respectively. The promoter sequences are in black; the gRNAa coding sequences are in green; and the 68 bp HDV ribozyme coding sequences are in blue. The primers used to generate these gRNA expression vectors are also included. The restriction enzymes Hind III, Bbs I and BamH I are highlighted in red.
