# Supplementary material for: Optimized CRISPR-Cas9 Genome Editing for Leishmania and Its Use To Target a Multigene Family, Induce Chromosomal Translocation, and Study DNA Break Repair Mechanisms
Source: mSphere. 2017 Jan 18;2(1):e00340-16. doi: 10.1128/mSphere.00340-16 (PMC5244264; doi:10.1128/mSphere.00340-16)
Supplement: DATA SET S5 [file sph001172218s5.docx]

**A.
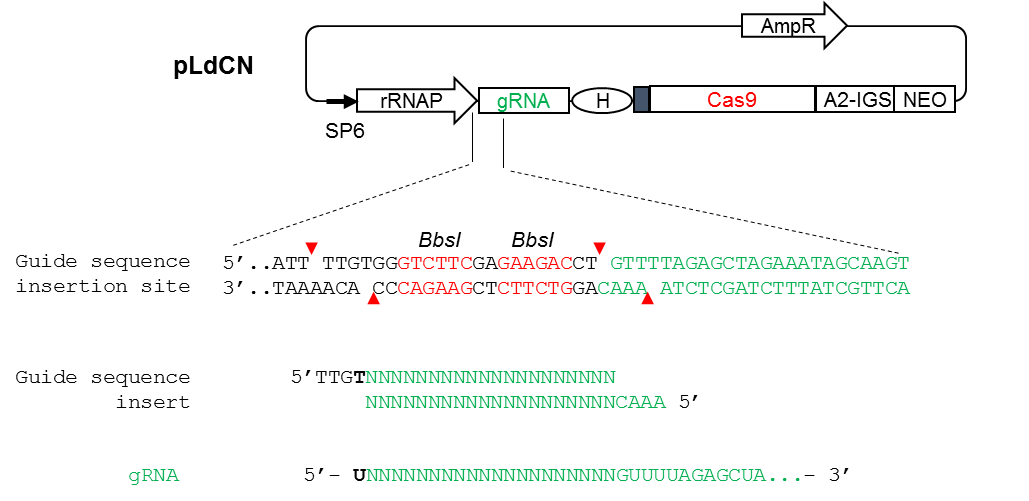
**

**B.** pLdCN (Xho I and BamH I fragment):

| 1 61 121 181 241 301 | CTCGAGTGTGAGTTATGAGGTCTGCGATTGACGTAGGAGTTGCAAGGGGGAGGGGGTATG  AACGGGGTGGGTAGAGCTTTTTTAGGTGGAAGTAGTGAGAGGGTGGGCTTGAGAGAATTT  GAGGTGTGTTCGTGATGTGTGGATCTTATCGGGGGCTCGGTTGAGTTTTTGGTTTGGTGA  TTTTG**T**GGGTCTTCGAGAAGACCTGTTTTAGAGCTAGAAATAGCAAGTTAAAATAAGGCT  AGTCCGTTATCAACTTGAAAAAGTGGCACCGAGTCGGTGCTTTTTTGGCCGGCATGGTCC  CAGCCTCCTCGCTGGCGCCGGCTGGGCAACATGCTTCGGCATGGCGAATGGGACGGATCC |
| --- | --- |

**C.** Primers used to generate pLdCN and pLdCH:

LdrRNApNde1 5’ GAGCAGCTGACATATGTGAGTTATGAGGTCTGCGA

LdrRNApXho1 5’ CCCGCTCGAGTGTGAGTTATGAGGTCTGCGA

pSPneoRHind3 5’ CTTGTTCAAGCTTGCGAATTCGAGC

**Data Set S5** *Leishmania* CRISPR vector pLdCN and its partial sequence. (A)Schematic of pLdCN and it guide sequence insertion site. rRNAP, *L. donovani* rRNA promoter; H, HDV ribozyme. The small ﬁlled black box represents the 92-bp pyrimidine track. The ﬁrst nucleotide U of gRNA is highlighted (black), as *L. donovani* rRNAP initiates transcription at the **T** residue site. The drawing is not to scale. (B) The partial sequence of pLdCN (XhoI and BamHI fragment), which includes the 180-bp rRNAP sequence in black and its transcription initiation site (**T**) in bold, the 82-bp Cas9 binding RNA coding sequence in green, and the 68-bp HDV ribozyme coding sequence in blue. The restriction enzymes XhoI, BbsI, and BamHI are highlighted in red. Note that the guide sequence insertion site for *Leishmania* CRISPR vector pLdCH is same as that for the pLdCN vector. (C) The primers used to generate pLdCN and pLdCH.
