# Supplementary material for: Optimized CRISPR-Cas9 Genome Editing for Leishmania and Its Use To Target a Multigene Family, Induce Chromosomal Translocation, and Study DNA Break Repair Mechanisms
Source: mSphere. 2017 Jan 18;2(1):e00340-16. doi: 10.1128/mSphere.00340-16 (PMC5244264; doi:10.1128/mSphere.00340-16)
Supplement: DATA SET S6 [file sph001172218s6.docx]

LdBPK_280580.1 | Leishmania donovani BPK282A1 | DNA repair protein RAD51, putative (RAD51) | genomic | Ld28_v01s1 reverse | (geneStart+0 to geneEnd+0) | length=1131

ATGCAGACCCGTTCTAAGGCCAAGGGTCGCCGTGGTCGTCCGTCGGCGCGGCCCTCTGAA

GAGGTTGAGGTTGTGGAGAGCCAGCCGCAGGAGGCCCTCCAGAATGAAGAGCAGGAGCCT

CGGCAGCAGCAGCAGCAGAGCACTGACATGGCTGAGCCGAACGCAAGTGGCTTTCGCGTT

ATCCAGATCTTGGAGAACTACGGCGTGGCGAGCTCGGATATCAAGAAGCTCATGGAGTGC

GGCTTTTACACGGTCGAGTCGGCGGCCTACGCTCCGAAGAAGGCCATCCTGGCAGTGAAG

GGGATCAGCGAGAACAAGGCCGAGAAAATTATGGCGGAGTGCGCCAAGCTGGTGCCGATG

GGGTTCACTTCCGCGGTCGCCTACCACGAGGCGCGCAAGGAGATCATTATGGTCACCACG

GGAAGCCGTGAGGTGGACAAGCTACTCGGCGGCGGCATCGAAACTGGGAGCATCACGGAG

CTCTTCGGAGAGTTCCGCACGGGCAAGACACAGCTCTGCCATACGCTGTGCGTGACGTGC

CAGCTGCCCATCTCTCAGGGTGGCGCGGAGGGCATGGCGCTCTATATCGACACCGAAGGC

ACCTTTCGCCCGGAGCGCCTCGTTGCCGTTGCGGAGCGGTACAAGCTGGACCCGGAGGAT

GTGCTCGCTAATGTGGCGTGTGCGCGTGCCTTCAACACGGATCACCAGCAGCAGCTGCTG

CTGCAGGCGTCTGCCATGATGGCCGAGAACCGCTTCGCGCTCATCGTCGTAGACTCTGCA

ACCGCTCTCTACCGCACAGACTACAGCGGCCGCAACGAGCTCGCGGCGCGGCAGATGCAC

CTCGGCAAATTCTTGCGCTCGCTGCACAACCTCGCCGAGGAGTACGGAGTGGCAGTGGTT

GTGACGAACCAAGTAGTTGCCAACGTGGACGGCTCCGCGCAGATGTTCCAGGCGGACTCT

AAGAAGCCGATTGGAGGCCACATTATGGCACACGCCTCGACGACGCGGCTTAGCCTGCGC

AAGGGTCGCGGCGAGCAGCGCATCATCAAGGTGTACGACTCTCCGTGCCTGGCCGAGGCT

GAGGCGATCTTTGGCATCTACGATGATGGCGTTGGTGACGCTCGGGATTGA

MQTRSKAKGRRGRPSARPSEEVEVVESQPQEALQNEEQEPRQQQQQSTDMAEPNASGFRV

IQILENYGVASSDIKKLMECGFYTVESAAYAPKKAILAVKGISENKAEKIMAECAKLVPM

GFTSAVAYHEARKEIIMVTTGSREVDKLLGGGIETGSITELFGEFRTGKTQLCHTLCVTC

QLPISQGGAEGMALYIDTEGTFRPERLVAVAERYKLDPEDVLANVACARAFNTDHQQQLL

LQASAMMAENRFALIVVDSATALYRTDYSGRNELAARQMHLGKFLRSLHNLAEEYGVAVV

VTNQVVANVDGSAQMFQADSKKPIGGHIMAHASTTRLSLRKGRGEQRIIKVYDSPCLAEA

EAIFGIYDDGVGDARD

Ld280580+ 5’TTGTGCCCATCTCTCAGGGTGGCG

Ld280580- CGGGTAGAGAGTCCCACCGCCAAA 5’

Ld280580donor:

5’ TGCCAGCTGCCCATCTCTCAGGGTGCAGTGAGTGAATTCTAGCGGGCGCGGAGGGCATGGCGCTCTATAT

DonorF 5’ CAGTGAGTGAATTCTAGCGG

DonorR 5’ CCGCTAGAATTCACTCACTG

Ld280580L 5’ CCCTCCAGAATGAAGAGCAG

Ld280580R 5’ CCATAATGTGGCCTCCAATC

PRODUCT SIZE: 894 bp

DonorF+Ld280580R 446 bp

Ld280580L+DonorR 488 bp

Q G G A

GCCCATCTCTCAGGGTGGCGCGGAGGGC

GCCCATCTCTCAAGGAGGAGGCGAGGGC

G

Ld280580donor2+: (sense worked)

5’ TGACGTGCCAGCTGCCCATCTCTCAAGGAGGAGGCGAGGGCATGGCGCTCTATATCGACA

Ld280580donor2: (antisense not working)

5’ TGTCGATATAGAGCGCCATGCCCTCGCCTCCTCCTTGAGAGATGGGCAGCTGGCACGTCA

Ld280580donor2F: 5’ CCATCTCTCAAGGAGGAGGC

Ld280580donor2R: 5’ CATGCCCTCGCCTCCTCCT

**Bleomycin expression cassette sequence:**

ATCTTCATCGGATCGGGTACCGAGCTCTCTCTTCTCCTTCCCCTCTCTCTCCTCTCTCCT CTCTCCAGAGCTCTCTCTTCTCCTTCCCCTCTCTCTCCTCTCTCCTCTCTCCAGAGCTCG AATTCATCGATGATATCAGATCCCCAACTAGATATCACCATGGCCAAGTTGACCAGTGCC GTTCCGGTGCTCACCGCGCGCGACGTCGCCGGAGCGGTCGAGTTCTGGACCGACCGGCTC GGGTTCTCCCGGGACTTCGTGGAGGACGACTTCGCCGGTGTGGTCCGGGACGACGTGACC CTGTTCATCAGCGCGGTCCAGGACCAGGTGGTGCCGGACAACACCCTGGCCTGGGTGTGG GTGCGCGGCCTGGACGAGCTGTACGCCGAGTGGTCGGAGGTCGTGTCCACGAACTTCCGG GACGCCTCCGGGCCGGCCATGACCGAGATCGGCGAGCAGCCGTGGGGGCGGGAGTTCGCC CTGCGCGACCCGGCCGGCAACTGCGTGCACTTCGTGGCCGAGGAGCAGGACTGA

280580BleF 5’ TGCCAGCTGCCCATCTCTCAGGGTGATCTTCATCGGATCGGGTAC

280580BleR 5’ ATATAGAGCGCCATGCCCTCCGCGCTCAGTCCTGCTCCTCGGCCA

**S. 6** *L. donovani* *RAD51* gene (LdBPK_280580.1) sequence and the sequences of gRNA guide, oligonucleotide donors and primers used to generate and detect *RAD51* mutants. The locations and directions of gRNA guide and primers in the *RAD51* gene are indicated and underlined with an arrow. Note: since the antisense oligo donor may hybridize to the corresponding gene transcripts (mRNA) which would prevent it from being used as an efficient template for the oligo donor directed repair, we were not able to generate the single amino acid substitution mutant with the antisense oligo donor Ld280580donor2 before using the sense oligo donor Ld280580donor2+.
